# The *Xanthomonas campestris* pv. *vesicatoria* Type-3 Effector XopB Inhibits Plant Defence Responses by Interfering with ROS Production

**DOI:** 10.1371/journal.pone.0159107

**Published:** 2016-07-11

**Authors:** Johannes Peter Roman Priller, Stephen Reid, Patrick Konein, Petra Dietrich, Sophia Sonnewald

**Affiliations:** 1 Department of Biology, Division of Biochemistry, Friedrich-Alexander-University Erlangen-Nuremberg, Erlangen, Germany; 2 Department of Biology, Division of Molecular Plant Physiology, Friedrich-Alexander-University Erlangen-Nuremberg, Erlangen, Germany; Indiana University, UNITED STATES

## Abstract

The bacterial pathogen *Xanthomonas campestris* pv. *vesicatoria* 85–10 (*Xcv*) translocates about 30 type-3 effector proteins (T3Es) into pepper plants (*Capsicum annuum*) to suppress plant immune responses. Among them is XopB which interferes with PTI, ETI and sugar-mediated defence responses, but the underlying molecular mechanisms and direct targets are unknown so far. Here, we examined the XopB-mediated suppression of plant defence responses in more detail. Infection of susceptible pepper plants with *Xcv* lacking x*opB* resulted in delayed symptom development compared to *Xcv* wild type infection concomitant with an increased formation of salicylic acid (SA) and expression of pathogenesis-related (*PR)* genes. Expression of *xopB* in *Arabidopsis thaliana* promoted the growth of the virulent *Pseudomonas syringae* pv. *tomato (Pst)* DC3000 strain. This was paralleled by a decreased SA-pool and a lower induction of SA-dependent *PR* gene expression. The expression pattern of early flg22-responsive marker genes indicated that MAPK signalling was not altered in the presence of XopB. However, XopB inhibited the flg22-triggered burst of reactive oxygen species (ROS). Consequently, the transcript accumulation of *AtOXI1*, a ROS-dependent marker gene, was reduced in *xopB*-expressing Arabidopsis plants as well as callose deposition. The lower ROS production correlated with a low level of basal and flg22-triggered expression of apoplastic peroxidases and the NADPH oxidase *RBOHD*. Conversely, deletion of *xopB* in *Xcv* caused a higher production of ROS in leaves of susceptible pepper plants. Together our results demonstrate that XopB modulates ROS responses and might thereby compromise plant defence.

## Introduction

Plants respond to bacterial pathogens with a vast array of defence responses. Recognition of the invading pathogen is a prerequisite for the activation of the defence system and the successful inhibition of bacterial propagation. Plants have employed a two-tier defence system to combat microbial invaders [1–3]. The first layer of defence is triggered by the recognition of pathogen associated molecular patterns (PAMPs) by surface-localised pattern recognition receptors leading to PAMP-triggered immunity (PTI). The second layer referred to as effector-triggered immunity (ETI) is activated by intracellular receptors that detect the presence and/ or activity of pathogen-derived effector molecules that have been secreted into the host cell. Generally, both perception systems elicit the activation of partially overlapping signalling cascades and defence responses [4]. However, responses are usually stronger and prolonged during ETI and often culminate in a kind of programmed cell death called hypersensitive response (HR) [5]. While PTI is sufficient to prevent multiplication of a wide range of non-adapted invaders, ETI is thought to be effective against adapted pathogens [2,3,5].

Most of our current knowledge of signalling events during PTI derives from the model plant *Arabidopsis thaliana* and relates to the perception of the PAMP flagellin [3,6,7]. The leucine rich repeat receptor kinase FLAGELLIN SENSING 2 (FLS2) recognises flg22, a conserved epitope present in the N-terminus of bacterial flagellin [8]. Binding of flg22 induces the instantaneous dimerization of the FLS2 receptor with the receptor-like kinase BRI1-associated kinase1 (BAK1) which is required to initiate signalling [9–11]. The subsequent phosphorylation of both proteins triggers downstream events such as the rapid influx of Ca^2+^ ions, the production of reactive oxygen species (ROS) and the activation of mitogen-activated protein kinase (MAPK) cascades [9,11].

The FLS2-BAK1 complex interacts with another, cytoplasmic receptor-like kinase termed Botrytis induced kinase 1 (BIK1) that becomes phosphorylated by BAK1 upon PAMP stimulation and is subsequently released from the complex to activate downstream processes. Recent work by Kadota et al. [12] Li et al. [13] uncovered that BIK1 directly interacts and phosphorylates the NADPH oxidase Respiratory burst oxidase homolog D (RBOHD). Beside this, RBOHD becomes activated by Ca^2+^ which directly binds to the EF-hand domains present in their N-terminal regions as well as by phosphorylation by Ca^2+^-dependent protein kinases (CDPKs) [14,15]. In response to flg22 treatment, RBOHD is the major enzyme for production of reactive oxygen species (ROS) [15–17]. Besides, the apoplastic class III peroxidases AtPRX33 and AtPRX34 are important for PAMP-mediated ROS production [18–20]. The rapid and transient production of ROS like superoxide anions and the more stable hydrogen peroxide (H_2_O_2_) is one of earliest responses starting a few minutes after PAMP treatment [21,22]. ROS can have a direct, toxic effect on the bacterial pathogens or act to confine microbial growth by cross linking plant cell wall proteins or by stimulating the production of phytoalexins. In addition, ROS function as signalling molecules by inducing defence gene expression and are involved in the redox control of proteins [21,22]. Pharmacological experiments revealed that the ROS burst is dependent on the Ca^2+^ influx [7,23]. The Ca^2+^ burst is a PTI hallmark [24,25] and is an important stimulus for many downstream responses. Thus, the activation of MAPKs and the subsequent reprogramming of gene expression that are observed upon PAMP stimulation are at least partially dependent on the Ca^2+^ burst [7,23,26,27]. Four CDPKs, namely CPK4, CPK5, CPK6 and CPK11, are also involved in transcriptional reprogramming upon flg22 stimulation [28]. MAPK- and CDPK-dependent signalling pathways can either act synergistically or independently to control expression of flg22-responsive genes [28]. Late responses upon PAMP treatment are amongst others the production salicylic acid (SA), the accumulation of antimicrobial secondary metabolites, the expression of *pathogenesis-related (PR) proteins* as well as the cell wall fortification by callose depositions [6].

Many Gram-negative pathogenic bacteria are capable to subvert PTI and/or ETI responses, by directly injecting type-3 effector proteins (T3Es) into the host cells using a type-3 secretion system (T3SS) [29–31]. The understanding of the *in planta* activities of T3Es, the elucidation of their modes of action and the identification of targeted host processes are major goals of pathophysiological research and is key to discover novel host-related defence components. The compatible interactions between *Pseudomonas syringae* pv. tomato (*Pst*) DC3000 and *A*. *thaliana* as well as between *Xanthomonas campestris pv*. *vesicatoria* (*Xcv*) (re-annotated as *X*. *euvesicatoria*) and tomato (*Solanum lycopersicum*) or pepper (*Capsicum annuum*) plants represent current model systems to unravel T3E functions [32,33]. Both bacteria inject at least 28 functional T3Es into host cells [34,35]. Although the functional analysis of T3Es is often hindered by their overlapping properties and functional redundancy, for some of them the mode of action has been uncovered (summarized in [29,30]). These studies revealed that some T3Es mimic eukaryotic proteins in structure and function, exhibit enzymatic activities and act for instance as proteases, E3-ubiquitin ligases, phosphotransferases or acetyltransferases [29,30]. Others T3Es function as transcriptional regulators or disrupt cellular structures to suppress plant defence. However, for many of these bacterial effectors their enzymatic activity and host targets remain to be unravelled.

We aim to understand the *in planta* function of the *Xcv* effector protein XopB. XopB has been identified as a T3SS-dependently secreted protein [36,37]. It does not belong to the conserved core group of T3Es and is absent in most *Xanthomonas* strains [35]. XopB shows high sequence similarity to known avirulence proteins such as HopD1 from *Pst* and AvrPphD of *P*. *syringae pv*. *phaseolicola* and to so far not investigated predicted T3E proteins of *X*. *fuscans* or *X*. *citri*. The protein consists of 613 amino acids, and does not possess any conserved domains or known linear motifs. In an early study, deletion of *xopB* in *Xcv* did not significantly alter symptom development and bacterial growth in susceptible pepper plants [36]. In contrast, Schulze et al. [37] found that infection with an *Xcv* Δ*xopB* strain led to less severe disease symptoms in susceptible plants than infection with the *Xcv* wild type. Ectopic expression of *xopB* in transgenic tobacco and tomato plants caused severe phenotypic alteration with malformed leaves and elicited cell death particularly in young and meristematic tissues [38]. Expression of *xopB* in yeast inhibited cell proliferation while transient expression in *N*. *benthamiana* resulted in the appearance of cell death symptoms [37,39]. XopB plays a role in PTI and ETI, as it suppresses HR-like cell death induced by several avirulence factors, but suppression of PTI and ETI might be caused by different mechanisms [37]. In PTI, XopB suppressed the flg22-mediated activation of the *AtNHL10* promoter, but did not influence the flg22-triggered phosphorylation of MAPKs. Together with its localization in Golgi vesicles and in the cytoplasm the authors suggested that XopB may inhibit PTI by interfering with vesicle transport processes [37].

In our previous work, we showed that XopB suppresses the induction of cell wall-bound invertase (cw-Inv) which is an important enzyme to fuel the sugar-enhanced defence responses [38]. In plants, an increased cw-Inv activity and/ or transcript accumulation has been observed in response to infection with different groups of pathogens including fungi, oomycetes, virus or bacteria [40,41]. Soluble sugars generated by cw-Inv activity provide energy to feed plant defence and act as signalling molecules to regulate gene expression and photosynthesis [41,42]. Moreover, cw-Inv activity is stimulated by PAMPs such as chitosan or a *Fusarium oxysporum lycopersici* elicitor preparation [43].

In this study, we analysed the role of XopB in PTI-related plant defence responses in more detail. To this end we generated transgenic *A*. *thaliana* plants expressing *xopB* under control of an ethanol-inducible promoter. Expression of *xopB* in transgenic *A*. *thaliana* plants led to a reduced accumulation of SA and a lower increase in *PR* marker gene expression as well as to an increased growth of the virulent *Pst* DC3000 strain indicating that XopB functions as a virulence factor. We show that XopB interferes with PTI responses by supressing the ROS burst after flg22-treatment in both *N*. *benthamiana* and *A*. *thaliana* plants. XopB contributes also in host plants to the suppression of defence responses most likely by interfering with the generation of ROS.

## Results

### XopB contributes to disease symptom development and alters SA content in susceptible pepper plants

**Fig 1:**
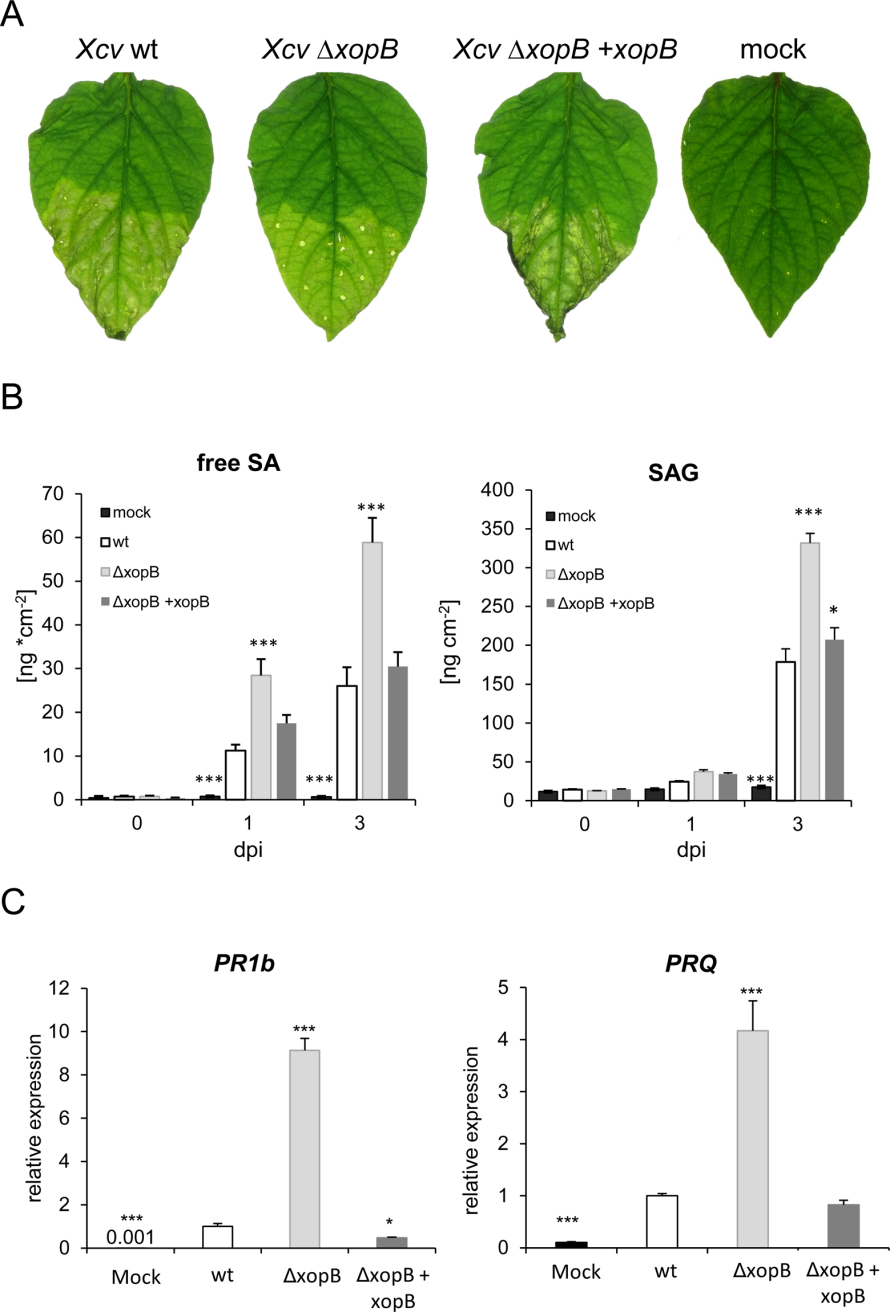
Impact of *xopB* on symptom development, SA content and expression of *PR* genes during *Xcv* infection of pepper leaves. Leaves of five week old susceptible pepper plants were inoculated with *Xcv* wild type (*Xcv* WT), *Xcv* Δ*xopB*, or a *xopB* deletion strain complemented with *xopB* (*Xcv* Δ*xopB* + *xopB*) under control of its own promotor at a concentration of 10^9^ cfu ml^-1^. All strains harbour the plasmid pRBB1-MCS5. As control, leaves were infiltrated with 10 mM MgCl_2_. **A)** Formation of disease symptoms in infected pepper leaves. Only the lower halves of the leaves were infiltrated. Pictures were taken 5 days post infection (dpi). Similar results were observed at least three times. **B)** Levels of free (free SA) and conjugated SA (SAG) in leaves, before, 1 and 3 days after infection with the *Xcv* strains indicated. Values represent the means +/- SE of four different samples. Statistically significant differences to *Xcv* wild type-infected leaves were determined using two-tailed t-test and are indicated by asterisks (***p<0.001; *p<0.05). The experiment was repeated with similar results. **C)** Transcript levels of *CaPR1b1* and *CaPRQ* were quantified by qPCR from samples taken 3dpi with the different *Xcv* strains. Values were normalized to *CaEF1alpha* and displayed relative to the expression level of *Xcv* wild type-infected leaves which were set to one. Values are means +/- SD of three independent samples each from a pool of two plants. Significant differences to *Xcv* wild type-infected leaves were calculated using t-test and are indicated by asterisks (***p<0.001; *p<0.05). Similar results were obtained in three independent experiments.

Infection of susceptible pepper plants with an *Xcv* Δ*xopB* deletion strain did not affect the timing of disease appearance and bacterial virulence in an early study [36]. However, a more recent study reported that deletion of *xopB* led to significantly reduced disease symptoms in infected pepper plants, while the bacterial growth *in planta* was not altered [37]. We also investigated the contribution of XopB to disease symptom development. Leaves inoculated with *Xcv* wild type developed severe necrotic lesions 5 days post infection (dpi), while those infected with the *Xcv* Δ*xopB* deletion strain displayed less severe signs of necrosis at this time point (Fig 1A). This delayed symptom development could be complemented by ectopic expression of *xopB* under control of its own promoter (Fig 1A). The complemented strain *Xcv* Δ*xopB* + *xopB* caused even stronger disease symptoms than the *Xcv* wild type strain which might be due to higher expression of *xopB* from the plasmid. No signs of disease were observed in Mock-infected control plants.

Next, we wanted to know whether XopB influences the accumulation of salicylic acid (SA) which is a plant hormone important for activation of plant defence [44]. Pepper plants were infected with *Xcv* wild type, *Xcv* Δ*xopB* and *Xcv* Δ*xopB* + *xopB* and the contents of free and glycosylated SA (SAG) were measured in leaf samples taken before, and 1 and 3 dpi. In *Xcv* wild type-infected leaves, an accumulation of free SA was visible at 1 dpi while SAG levels were clearly increased only 3 dpi (Fig 1B). Infection with the *Xcv xopB* deletion strain resulted in an about 2-fold higher accumulation in the amounts of free and conjugated SA as compared to *Xcv* wild type-infected leaves. In contrast, infection with the complementation strain *Xcv* Δ*xopB* + *xopB* induced an increase in SA-levels similar to *Xcv* wild type. These results suggest that the presence of XopB causes a suppression of plant defence responses.

This conclusion was further confirmed by measuring transcript abundance of the defence genes *CaPR1b1* (*pathogenesis- related protein 1b1*) and *CaPRQ* (*chitinase*) by quantitative real-time RT-PCR (qPCR). Expression of both genes is regulated by SA while *CaPRQ* is also stimulated by sugars [45,46] and is suppressed by XopB as shown by northern blotting in previous work [38].

In *Xcv* Δ*xopB-*infected leaves, the mRNA levels for *CaPR1b1* and *CaPRQ* were 9-fold and 4-fold higher, respectively, compared to *Xcv* wild type-infected leaves (Fig 1C), consistent with the elevated SA pool. Infection with the *xopB* complementation strain induced expression of both *PR* genes to a similar extent as *Xcv* wild type (Fig 1C).

Together, these findings demonstrate that XopB suppresses SA accumulation and SA-dependent defence gene expression in pepper plants during *Xcv* infection.

### Inducible expression of *xopB* causes cell death in transgenic Arabidopsis plants

In order to investigate the function of XopB *in planta* in more depth we generated transgenic *A*. *thaliana* expressing *xopB* under the control of an ethanol-inducible promoter [47]. To this end, the open reading frame was cloned and inserted into the Bin19-derived vector p35S::alcR as described [38] (S1A Fig). Several kanamycin-resistant, *xopB*-expressing plants were obtained. The presence of *xopB* was analysed by northern blotting upon floating of leaf discs with 0.2% (v/v) ethanol (S1B Fig). Four lines (10, 12, 16, 27) were selected to determine the number of T-DNA insertions by southern blotting (S1C Fig). While lines 16 and 27 harbour at least two T-DNA insertions, lines 10 and 12 carry single T-DNA insertions and were therefore used for further experiments.

**Fig 2:**
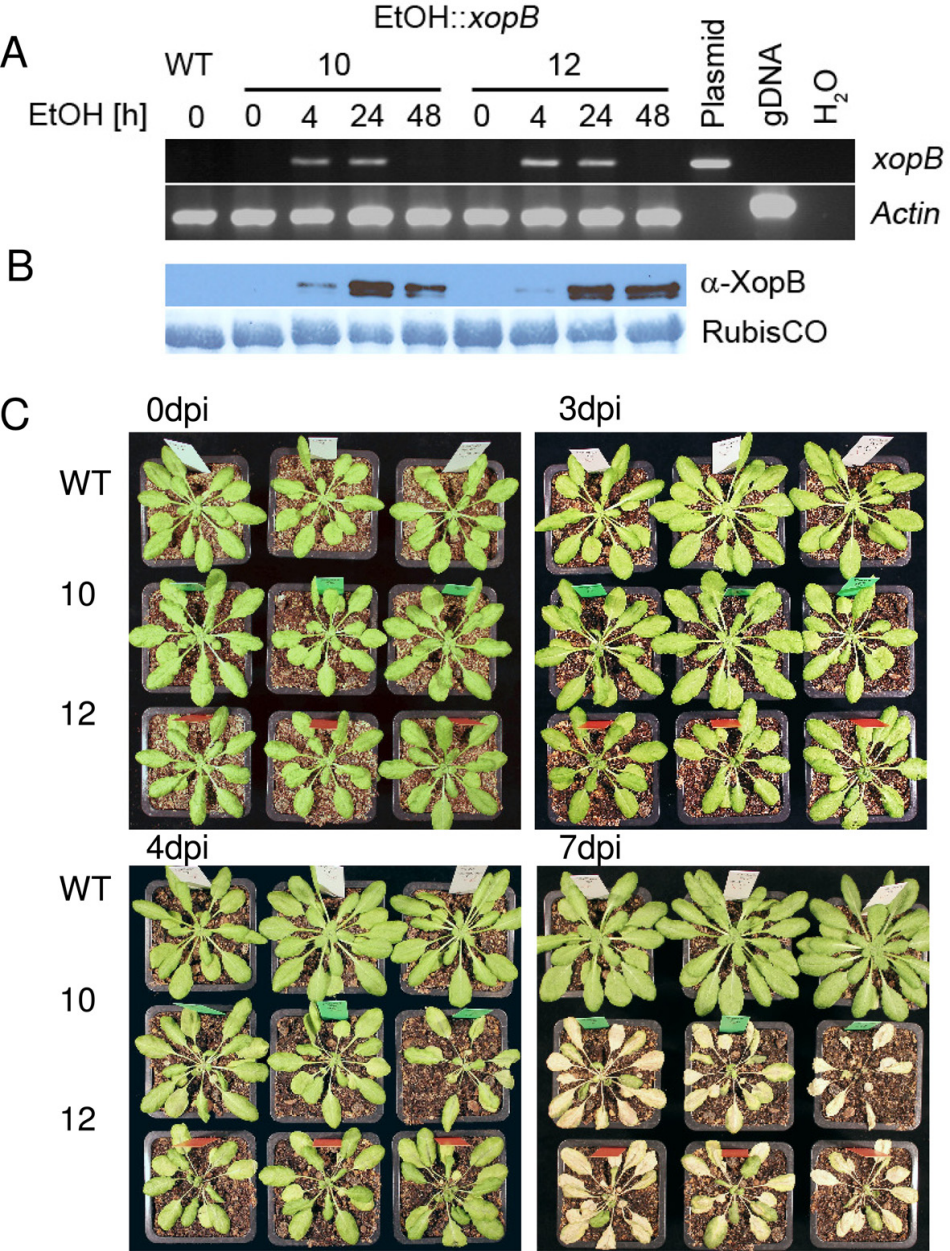
Inducible expression of *xopB* in transgenic *A*. *thaliana* plants causes severe phenotypic changes. Arabidopsis wild type and two independent *xopB*-expressing transgenic plants (EtOH::*xopB*, lines 10 and 12) were analysed. Expression of *xopB* was induced by watering ca. five week old plants with 10 ml 1% (v/v) EtOH. **A)** Analysis of *xopB*-specific transcript accumulation by RT-PCR in the transgenic lines 10 and 12 before and 4 h, 24 h and 48 h after ethanol application. As reference, *Actin*-specific mRNA levels are shown. The binary plasmid (Plasmid) which was used for *A*. *thaliana* transformation was included as positive control, genomic DNA (gDNA) from wild type to exclude contamination with genomic DNA and water (H_2_O) as negative control. **B)** Analysis of XopB protein accumulation upon induction of *xopB* expression in the transgenic lines (10, 12) by western blotting with a XopB-specific antibody at time points described in (A). The amido black stained RubisCO-band is shown as loading control. **C)** Phenotypic changes in transgenic Arabidopsis plants caused by *xopB* expression compared to wild type. Shown are three plants each before watering plants with ethanol (0dpi) and 3, 4 or 7 dpi.

Approximately 6-week old, soil-grown plants were watered with 10 ml 1% EtOH and expression of *xopB* was verified by RT-PCR and western blotting using a XopB-specific antibody (Fig 2A and 2B). Both the *xopB*-specific transcript and the protein were already detectable 4h after ethanol induction. The *xopB*-specific mRNA was not detectable after 48h, while the corresponding protein was still present at this time point (Fig 2A and 2B).

After induction of *xopB* expression by ethanol the transgenic plants stopped growing and leaves developed first chlorotic spots after about three days. In particular, older leaves residing directly above the soil were affected at this time point (Fig 2C). In the following hours also upper leaves developed chlorosis. Subsequently, chlorotic leaves became necrotic (day 4) and died back (day 7). Appearance of cell death symptoms started from the petioles and spread over the midrib into the leaf lamina (Fig 2C). In contrast, no phenotypic differences between wild type and transgenic plants were observed before treatment with ethanol.

These results show that the T3E protein XopB causes severe phenotypic changes leading to cell death when ectopically expressed in *A*. *thaliana* suggesting that XopB may interfere with plant metabolism [38] and / or signalling.

### Expression of *xopB* in Arabidopsis supports in planta growth of *Pst* DC3000

**Fig 3:**
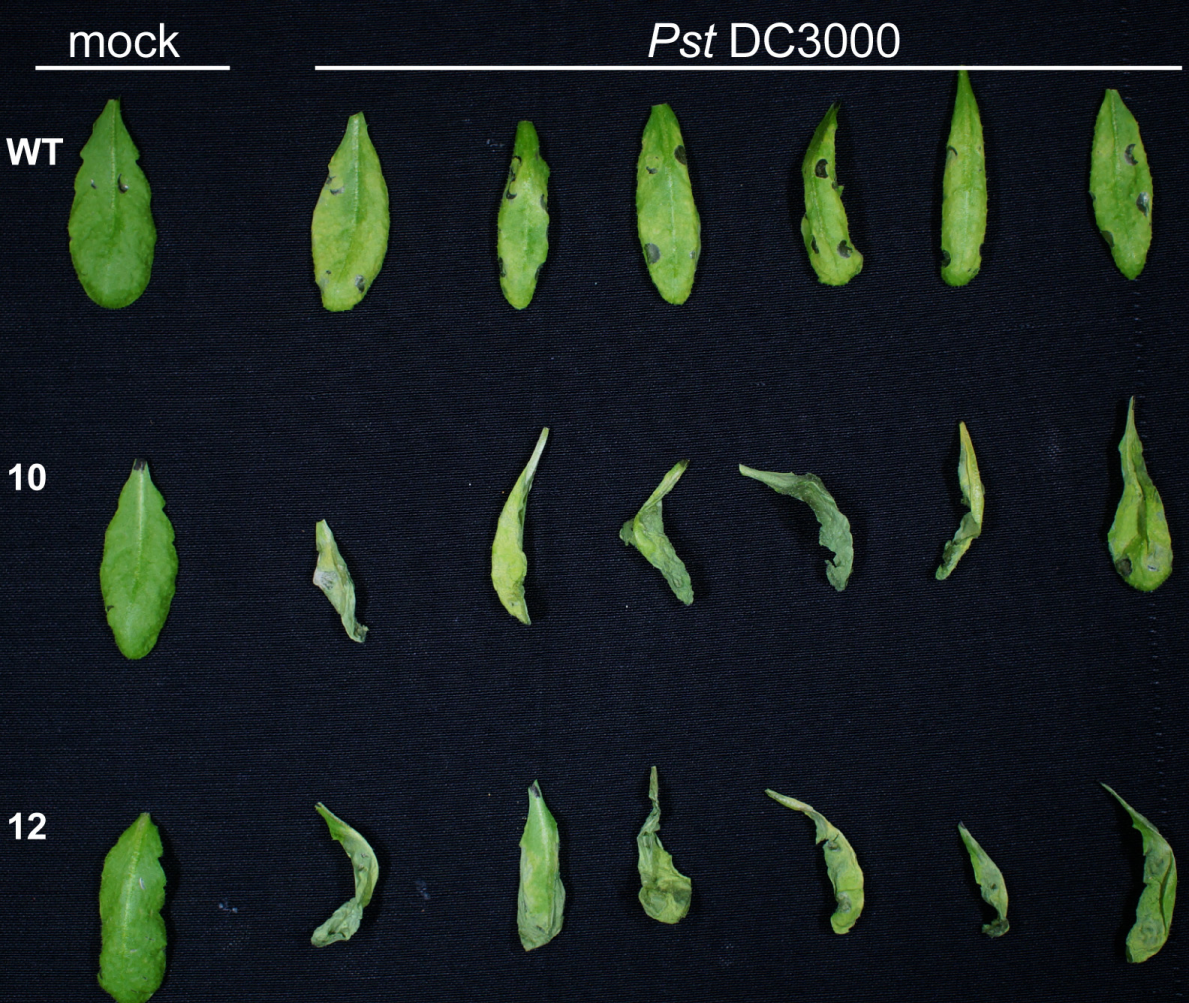
*xopB* expression accelerates development of disease symptoms in *A*. *thaliana* plants after *Pst* DC3000 infection. *A*. *thaliana* wild type and x*opB-*expressing plants (lines 10 and 12) were watered with 10 ml 1% EtOH each. Eighteen hours later they were infiltrated with *Pst* DC3000 adjusted to a bacterial titre of 5*10^5^ cfu ml^-1^. As control plants were infiltrated with 10 mM MgCl_2_ (Mock). Disease symptoms were documented at 3 dpi. Six infected and one representative mock-infiltrated leaves are shown. The experiment was performed three times with similar results.

**Fig 4:**
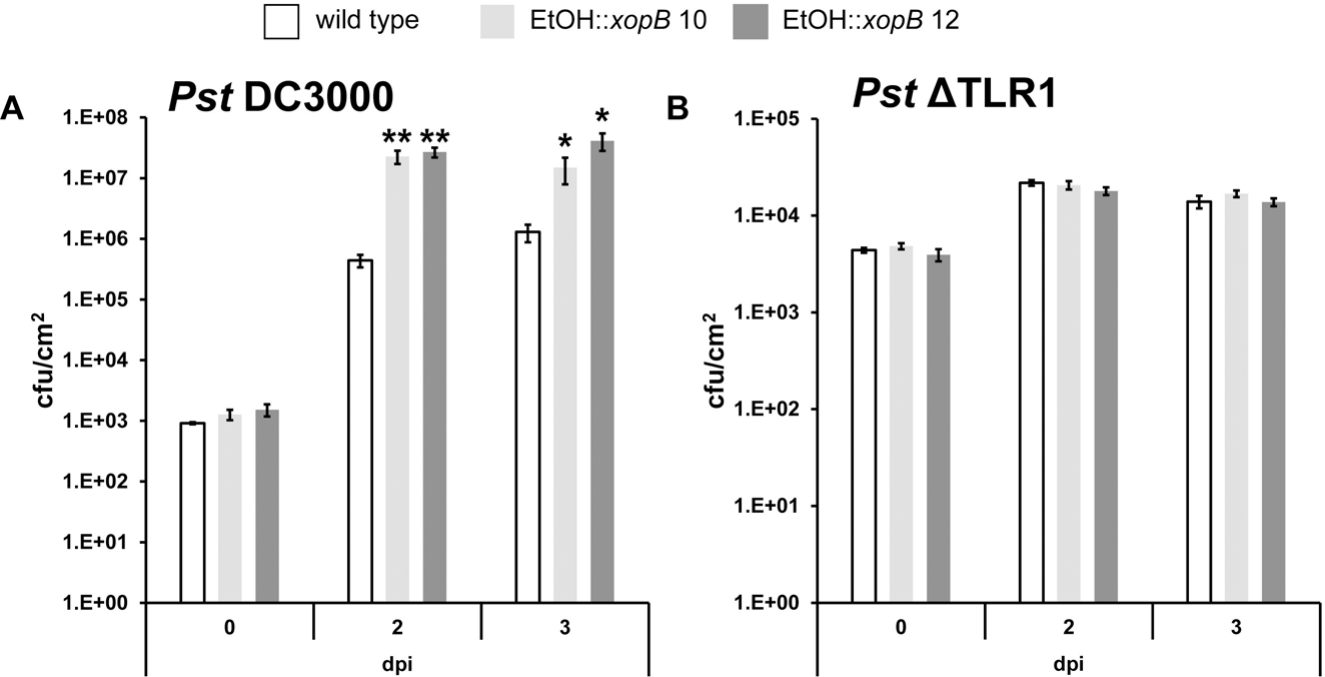
Expression of *xopB* in *A*. *thaliana* supports the *in planta* growth of the *Pst* DC3000 strain, but not of the *Pst* ΔTLR1 strain. *A*. *thaliana* wild type and transgenic EtOH::*xopB* plants (lines 10 and 12) were watered with 10 ml 1% EtOH 18 h before infiltration with (**A**) the *Pst* DC3000 strain or (**B**) the TTSS-deficient mutant strain (*Pst* ΔTLR1) to monitor the bacterial growth *in planta*. *Pst* DC3000 and *Pst* ΔTLR1 were infiltrated using a needle-less syringe with a bacterial density of 5*10^5^ cfu ml^-1^ or 10^6^ cfu ml^-1^, respectively. Bacterial titres were determined at indicated time points from 3 biological replicates (3 pools out of 6 plants). Values represent means +/- SE. Statistically significant differences between the wild type and the EtOH::*xopB* lines 10 and 12, respectively, were determined using a two-tailed t-test assuming normal distribution and are indicated by asterisks. Similar results were obtained in three independent experiments.

In order to investigate whether XopB has an impact on bacterial virulence, leaves of wild type and *xopB*-expressing plants were infected with *Pst* DC3000 18 h after induction of *xopB* expression. Compared to *Pst* DC3000-infected wild type leaves, which displayed only mild chlorosis, leaves of infected *xopB-*expressing plants were already severely damaged 3 dpi and died earlier than Mock-infected control leaves (Fig 3). Next, the *in planta* growth of *Pst* DC3000 was determined in order to analyse if the visible increase in disease symptom development caused by *xopB* expression was accompanied by an altered bacterial titre. Two and three days after infection, the *xopB*-expressing lines contained a more than one order of magnitude higher number of bacteria (Fig 4A) than wild type plants, as expected from the observed accelerated disease symptom development (Fig 3).

In addition to *Pst* DC3000, wild type and transgenic plants were infected with the TTSS-deficient mutant *Pst* ΔTLR1 to investigate whether basal defence is significantly dampened by XopB. The *in planta* growth of the *Pst* ΔTLR1 strain, however, was not altered in the *xopB-* expressing lines as compared to wild type plants (Fig 4B). This suggests that the heterologous expression of XopB is not sufficient to promote the *in planta* growth of the TTSS-deficient *Pst* strain, but rather acts synergistically with T3Es translocated by *Pst* DC3000 to suppress plant defence responses and to support bacterial propagation *in planta*.

### XopB modulates SA accumulation and SA-dependent gene expression in Arabidopsis

**Fig 5:**
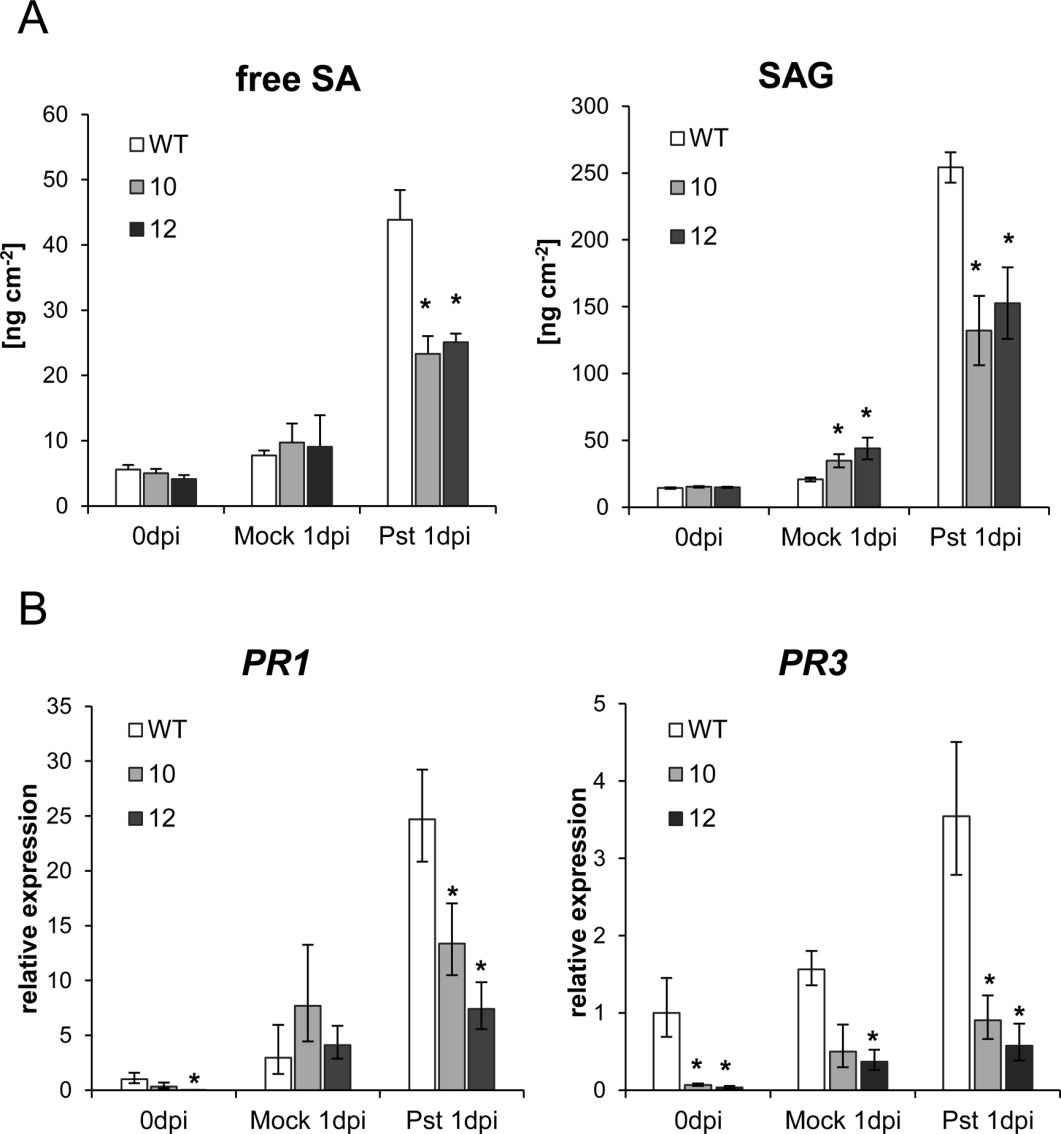
Expression of *xopB* causes reduced SA accumulation and expression of SA-dependent *PR* genes. *A*. *thaliana* wild type and *xopB*- expressing plants (lines 10 and 12) were watered with 10 ml 1% EtOH 18 h before infiltration with the virulent *Pst* strain DC3000 at a bacterial density of 2.5*10^6^ cfu ml^-1^ or with 10mM MgCl_2_. **A)** Contents of free (free SA) and conjugated SA (SAG) were quantified with HPLC before (0 dpi) and 1 day after infection (1 dpi). Values represent the means +/- SE of two different experiments each with four independent replicates. Statistically significant differences in the SA/ SAG contents between the wild type and the EtOH::*xopB* lines 10 and 12, respectively, were determined using a two-tailed t-test assuming normal distribution and are indicated by asterisks (*p<0.05). **B)** Total RNA was extracted before (0 dpi) and 1 dpi and reverse transcribed into cDNA. Abundances of *AtPR1-* and *AtPR3-*specific transcripts were determined by qPCR, normalized to *AtTUB4* and displayed relative to the expression level in wild type plants. Values are means +/- SD of three independent replicates each measured in triplicates. Statistically significant differences compared to wild type plants were determined using two-tailed t-test and are indicated by asterisks (*p<0.05). The experiment was repeated twice with similar results.

Since XopB suppresses SA-responses in *Xcv*-infected pepper plants we tested whether this was also the case in the *Pst* DC3000- infected Arabidopsis plants. To this end, wild type and transgenic plants were infected with *Pst* DC3000 18 h after ethanol-watering to induce *xopB* expression. Leaf samples were taken before infection and 1 dpi and subjected to HPLC analysis. In response to *Pst* DC3000, Arabidopsis wild type leaves accumulated substantial amounts of free and conjugated SA (Fig 5A). The increase in free and glycosylated SA contents was about half of that in both *xopB*-expression lines (Fig 5A) indicating that the presence of XopB compromised their accumulation. Before infection, meaning 18 h after induction of *xopB* expression, XopB did not significantly influence the amounts of SA and SAG. However, there was a significant increase in the content of SAG in Mock-infected *xopB-*expressing plants as compared to the Mock-infected wild type plants (Fig 5A) which may indicate increased stress level due to expression of *xopB* and wounding.

In addition to the SA levels the transcript amounts of the SA-dependent *PR* genes *AtPR1* and *AtPR3* were determined by qPCR. Consistent with the strong SA/SAG accumulation in wild type plants, there was a significantly increased expression of both genes after *Pst* DC3000 infection (Fig 5B). Relative to Mock-infected wild type plants, mRNA levels of *AtPR1* and *AtPR3* were 8- and 2.3-fold higher in *Pst* DC 3000-infected plants, respectively. Transcript abundance of *AtPR1* and *AtPR3* was clearly reduced in *xopB*-expressing lines before infection (Fig 5B) suggesting that the heterologous expression of XopB suppresses plant defence responses. Similar to the SAG pool, an increase in the transcript amounts of *AtPR1* and *AtPR3* was detected in the Mock-infiltrated transgenic plants, while infection with *Pst* DC3000 caused only a slight further increase in mRNA levels of *AtPR1* and *AtPR3* and their absolute levels were significantly lower than in *Pst* DC3000-infected wild type plants (Fig 5B).

### XopB differentially modulates expression flg22-responsive marker genes

Since it has been shown that XopB suppresses PTI when transiently expressed in Arabidopsis protoplasts [37], we analysed whether PTI is also attenuated in *xopB*-expressing *A*. *thaliana* plants. Expression of flg22-responsive markers genes was quantified, including *AtFRK1* and *WRKY22* as MAPK-specific targets and *AtPHI1* as a CDPK-dependently regulated gene [26,28]. In addition, *AtNHL10* was chosen as a gene controlled synergistically by both pathways [26,28]. The *fls2* mutant served as a control, because it does not respond to flg22 [8].

**Fig 6:**
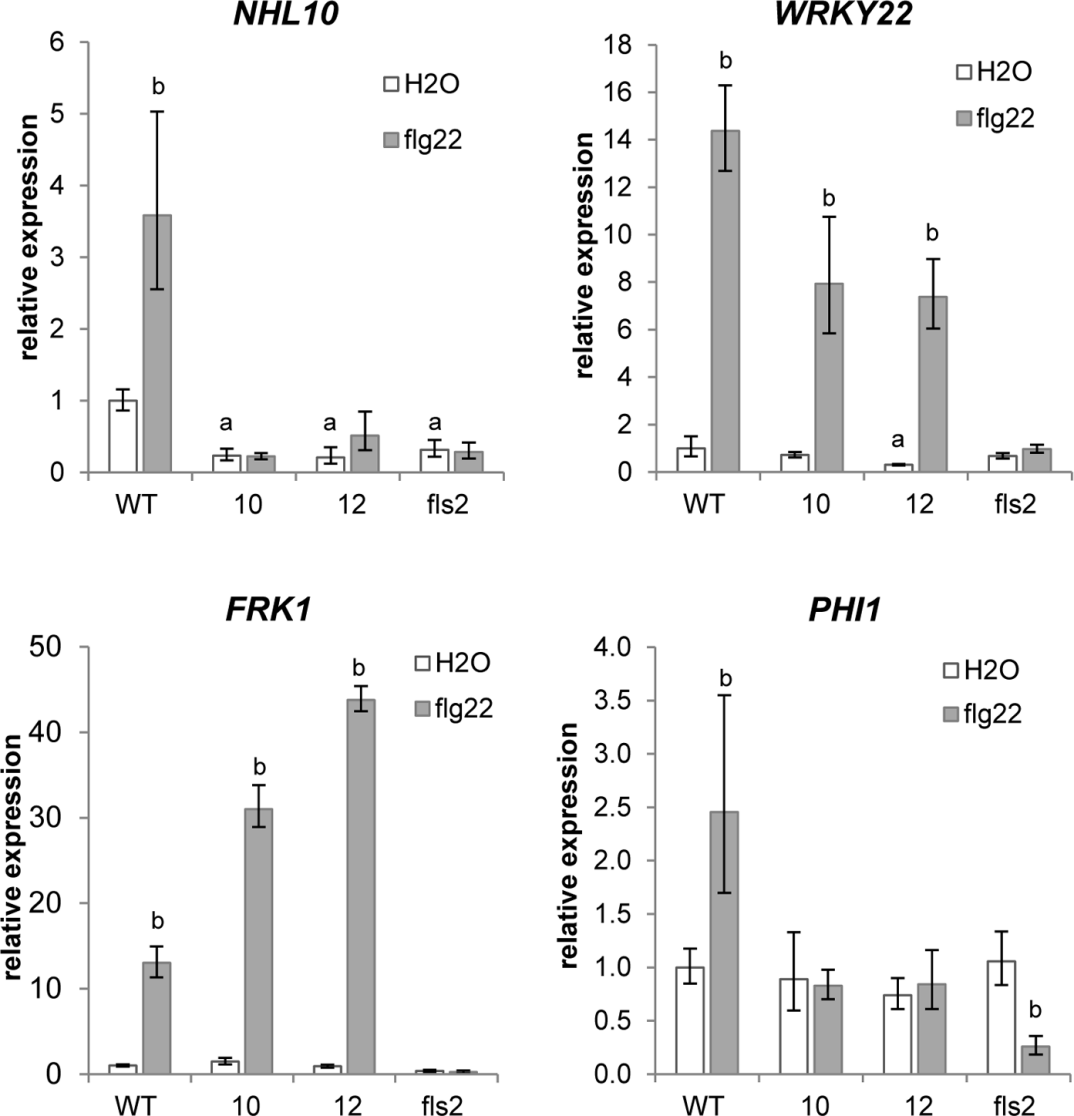
Expression of *xopB* in Arabidopsis differentially modulates expression of flg22-responsive genes. *A*. *thaliana* wild type (WT) and *xopB*-expressing lines 10 and 12 were watered together with *fls2* mutant plants with 10 ml 1% EtOH. After 18 h leaves were infiltrated with 1 μM flg22 or deionized water. Leaf material was harvested 60 min after treatment. Total RNA was isolated and reverse transcribed into cDNA. mRNA accumulation of *AtNHL10*, *AtWRKY22*, *AtFRK1*, and *AtPHI1* was measured by qPCR. Expression of *AtTUB4* was used to normalize the expression values. Expression levels are shown relative to the mean of water-treated wild type samples. Values are means +/- SD of three independent replicates. Statistical differences (p<0.05) were determined (a) between water-treated wild type and the transgenic or fls2 plants and (b) between water- and flg22-treated plants, respectively, using two-tailed t-test assuming normal distribution. Similar results were obtained in two independent experiments.

Expression of *AtNHL10* was 3.8-fold induced by flg22 treatment in wild type plants, while its expression was not flg22-inducible in the *fls2* mutant (Fig 6, S1 Table). Similarly to the *fls2* mutant, *AtNHL10* mRNA level was not or only slightly increased in response to the flg22 treatment in both *xopB*-expressing lines. Remarkably, expression of *AtNHL10* was significantly diminished in water-treated transgenic and *fls2* plants as compared to wild type plants (Fig 6). These results support the findings that XopB suppresses both the basal and the flg22-mediated activation of the *NHL10* promoter [37].

The mRNA levels of *AtWRKY22* were also lower in water-treated transgenic plants as compared to the wild type, but in contrast to *AtNLH10*, *AtWRKY22* was strongly induced in response to flg22 treatment in wild type as well as in *xopB-*expressing plants (Fig 6). Thus, flg22 treatment elicited an about 15-fold increase in *AtWRKY22* transcript amounts in wild type plants and a 14- and 25-fold induction in *xopB-*expressing lines 10 and 12, respectively (S1 Table). As expected, there was no flg22-mediated increase in *AtWRKY22* mRNA amounts in the *fls2* plants (Fig 6, S1 Table). *AtFRK1* was similarly expressed in non-stimulated *xopB*-expressing and wild type plants. Flg22 treatment resulted in a significant increase in *AtFRK1* transcript abundance in wild type and both transgenic plants, but not in the *fls2* mutant. As for *AtWRKY22*, the flg22-mediated increase in *AtFRK1* expression was even higher in the *xopB* line 12 as compared to the wild type (Fig 6, S1 Table). These results suggest that the flg22-elicited induction of the MAPK-dependent genes *AtWRKY22* and *AtFRK1* was not affected or even stimulated by XopB. In contrast, the flg22-induced transcriptional activation of *AtNHL10* was hampered by XopB. Hence, it is tempting to speculate that in the *xopB*-expressing plants the CDPK-dependent rather than the MAPK-dependent signalling pathway is influenced. In fact, expression of the CDPK-dependent gene *AtPHI1* was not increased by flg22 treatment in the *xopB*-expressing lines, but was 2.6-fold increased in wild type plants (Fig 6). Moreover, *AtPHI1* expression was not altered by XopB in water-treated plants, but was decreased in the *fls2* mutant after flg22 treatment.

### XopB interferes with flg22-mediated ROS burst and inhibits callose deposition

The results so far suggested that XopB might inhibit the CDPK- dependent branch of flg22- responsive gene expression, but not the MAPK-dependent one. This supports findings by Schulze et al. [37] that XopB did not alter the phosphorylation of MAPKs. Several publications showed that the calcium response is upstream of two separate signalling pathways; one leading to activation of MAPK and the other to ROS production [7,23,48]. Since MAPK signalling appeared not to be addressed by XopB, we investigated whether it affects the flg22-mediated ROS burst.

**Fig 7:**
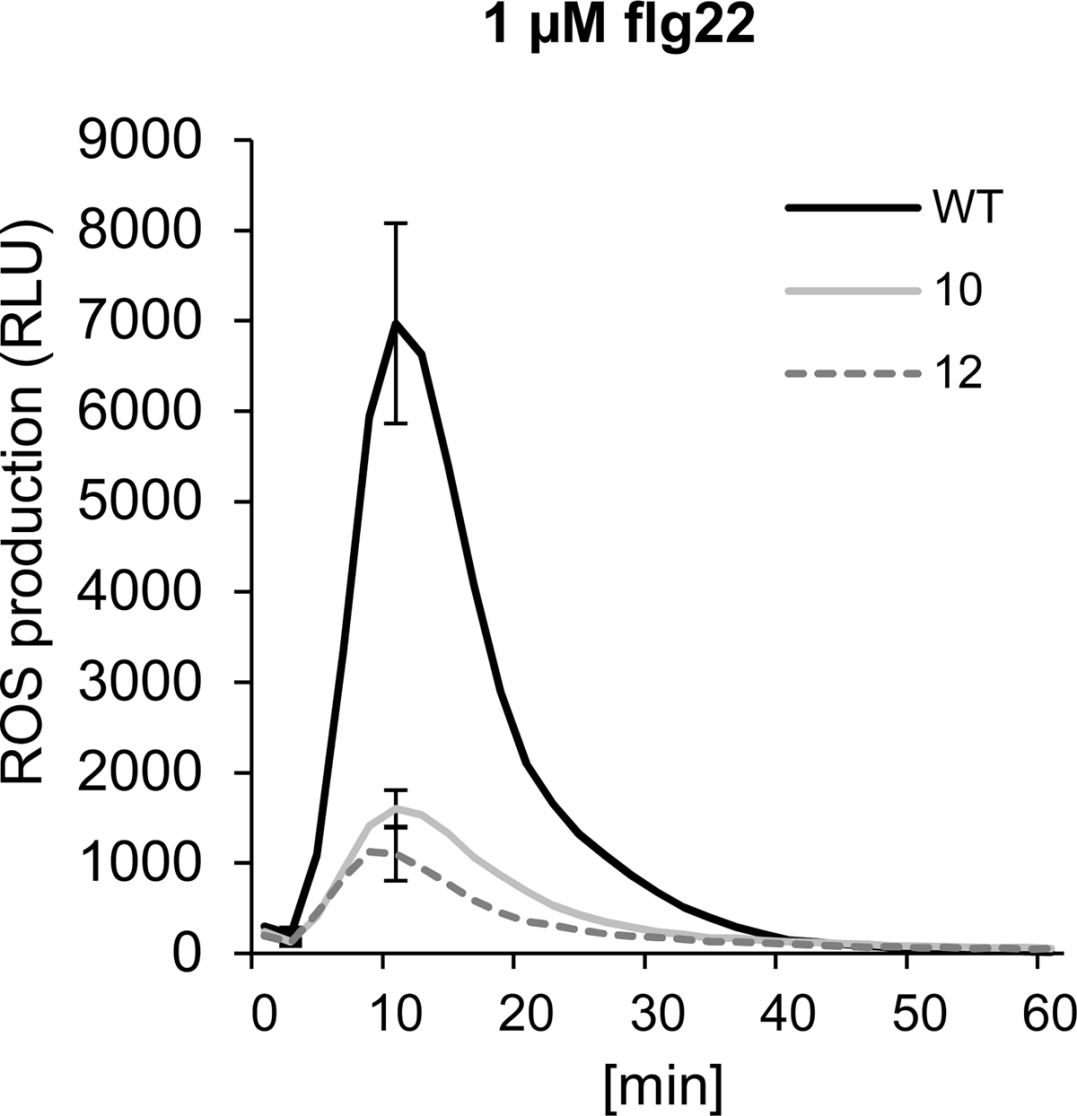
XopB suppresses the flg22-mediated ROS burst *A*. *thaliana*. ROS production (RLU, relative luminescence units) was measured in wild type (black line) and transgenic *A*. *thaliana* lines 10 (light grey line) and 12 (dark grey line) upon stimulation with 1 μM flg22. All leaf discs were incubated in 0.2% EtOH for 18 h to induce *xopB* expression. Values are means +/- SE of 8 independent samples. Statistically significant differences were determined using two-tailed t-test assuming normal distribution. Statistically significance between wild type and the transgenic lines are indicated by asterisks (p<0.05). The experiment was repeated three times with similar results.

To this end, leaf discs from *xopB*-expressing and *A*. *thaliana* wild type plants were stimulated with flg22 and the time-dependent accumulation of ROS was measured. As expected, flg22 elicited a strong, but transient increase in ROS in wild type plants (Fig 7). Interestingly, leaf discs from *xopB*-expressing lines 10 and 12 showed a 70% and 80% lower ROS production compared to wild type plants, respectively (Fig 7) indicating that XopB suppresses the flg22-triggered ROS generation. To verify this finding, we measured the ROS burst after flg22 treatment in *N*. *benthamiana* leaves transiently expressing *xopB* or an empty vector. Like in Arabidopsis, expression of *xopB* also abolished the flg22-induced ROS burst in this experimental system (S2 Fig).

To investigate whether the altered ROS production in *xopB*-expressing lines is reflected by altered expression of ROS-related genes we determined the transcript abundance of *AtRBOHD*, *AtPRX33*, *AtPRX34* and of *OXIDATIVE SIGNAL-INDUCIBLE1* (*AtOXI1*) 30 min after flg22 treatment. Control plants were treated with water and the *fls2* mutant was again included.

**Fig 8:**
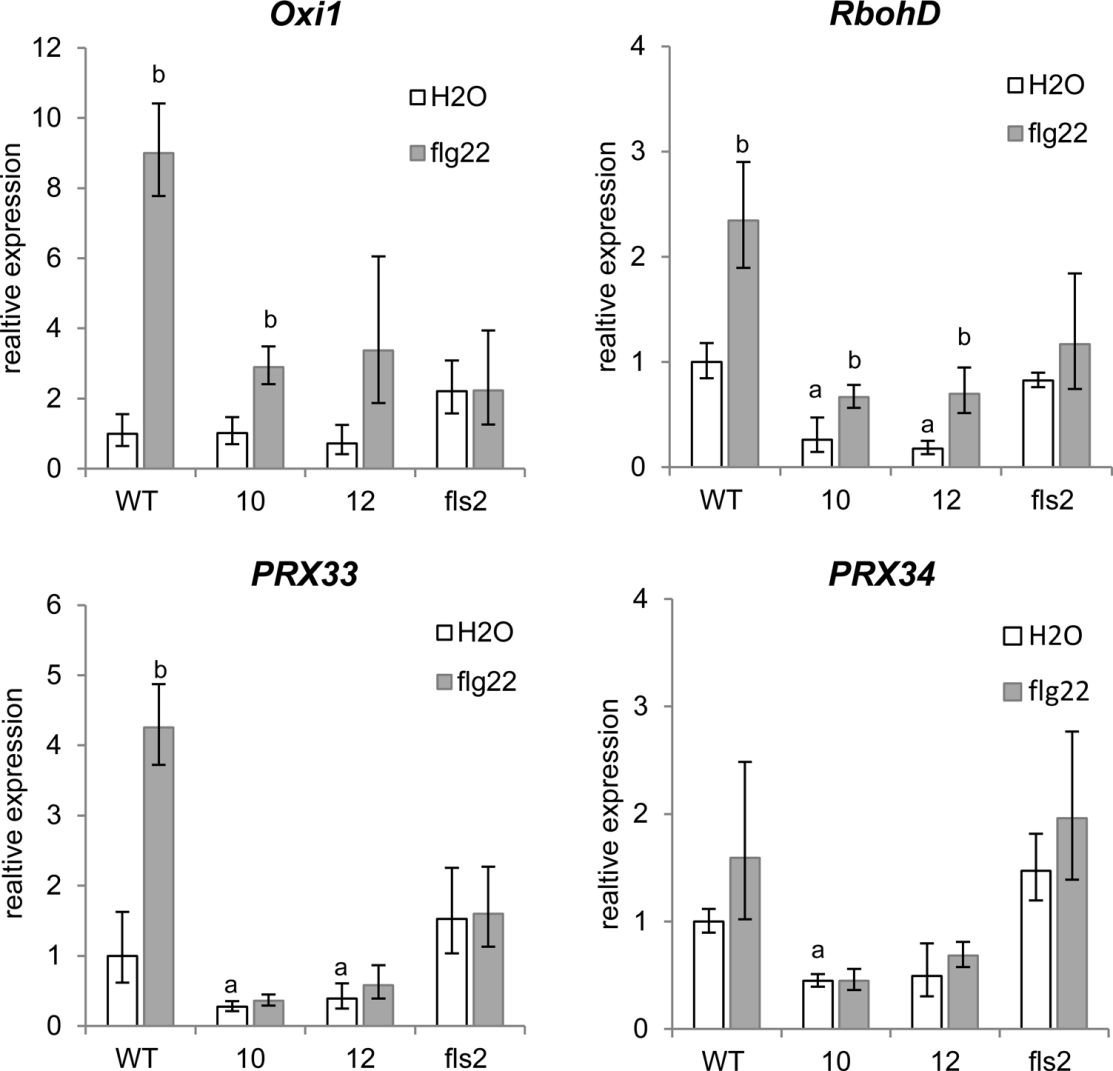
Expression of *xopB* in Arabidopsis alters expression of ROS-responsive and ROS-producing enzymes. *A*. *thaliana* wild type (WT), *xopB*-expressing lines 10, 12 and *fls2* mutant plants were watered with 10 ml 1% EtOH. After 18 h leaves were infiltrated with 1 μM flg22 or deionized water as a control. Samples were taken after 30min. Total RNA was isolated and reverse transcribed into cDNA. Transcript accumulation of *AtOXI1*, *AtRBOHD*, *AtPRX33*, and *AtPRX34* was measured by qPCR. Expression of *AtTUB4* was used to normalize the expression of each sample. Expression levels are shown relative to the water-treated wild type. Values are means +/- SD of three independent replicates. Statistical differences (p<0.05) were determined (a) between water-treated wild type and the transgenic or fls2 plants and (b) between water- and the flg22-treated plants, respectively, using two-tailed t-test assuming normal distribution. Similar results were obtained in two independent experiments.

Expression of *AtOXI1* which was shown to be transcriptionally activated by H_2_O_2_ [49] was similar in wild type and transgenic water-treated plants, but was ca. 2-fold higher in *fls2* controls compared to wild type. As expected, flg22 did not increase expression of *AtOXI1* in the *fls2* mutant, but elicited an about 9-fold increase in mRNA abundance in wild type plants (Fig 8). In comparison, there was only a 3-fold induction of *AtOXI1* by flg22 in the *xopB*-expressing plants (Fig 8), in accordance with the diminished ROS production in these lines. The mRNA abundance of *AtRBOHD* was already 4- to 6-times lower in the water-treated *xopB-*expressing lines compared to wild type controls (Fig 8), indicating an interference of XopB with the redox homeostasis in the absence of PAMPs. Upon flg22 stimulation *AtRBOHD* mRNA level increased 2.7-fold in wild type plants, but no significant induction was detectable in the *fls2* mutant. Although the fold-change compared to the control situation was similar in *xopB*-expressing lines and wild type (2.6- and 4.0-fold in lines 10 and 12, respectively), the absolute *AtRBOHD* transcript amounts were lower in both *xopB-*expressing lines upon flg22 treatment compared to those in wild type in the absence of the PAMP. The mRNA abundance of *AtPRX33* and *AtPRX34* were also 2- to 4-times lower in water-treated transgenic plants compared to wild type plants supporting the hypothesis that XopB may constitutively affect expression of ROS producing enzymes. Expression of both peroxidases was hardly induced by flg22 in the *xopB*-expressing lines as well as in the *fls*2 mutant. The amount of *AtPRX33* mRNA was clearly increased in flg22-treated wild type plants, while expression of *AtPRX34* was not significantly up-regulated in wild type plants (Fig 8).

**Fig 9:**
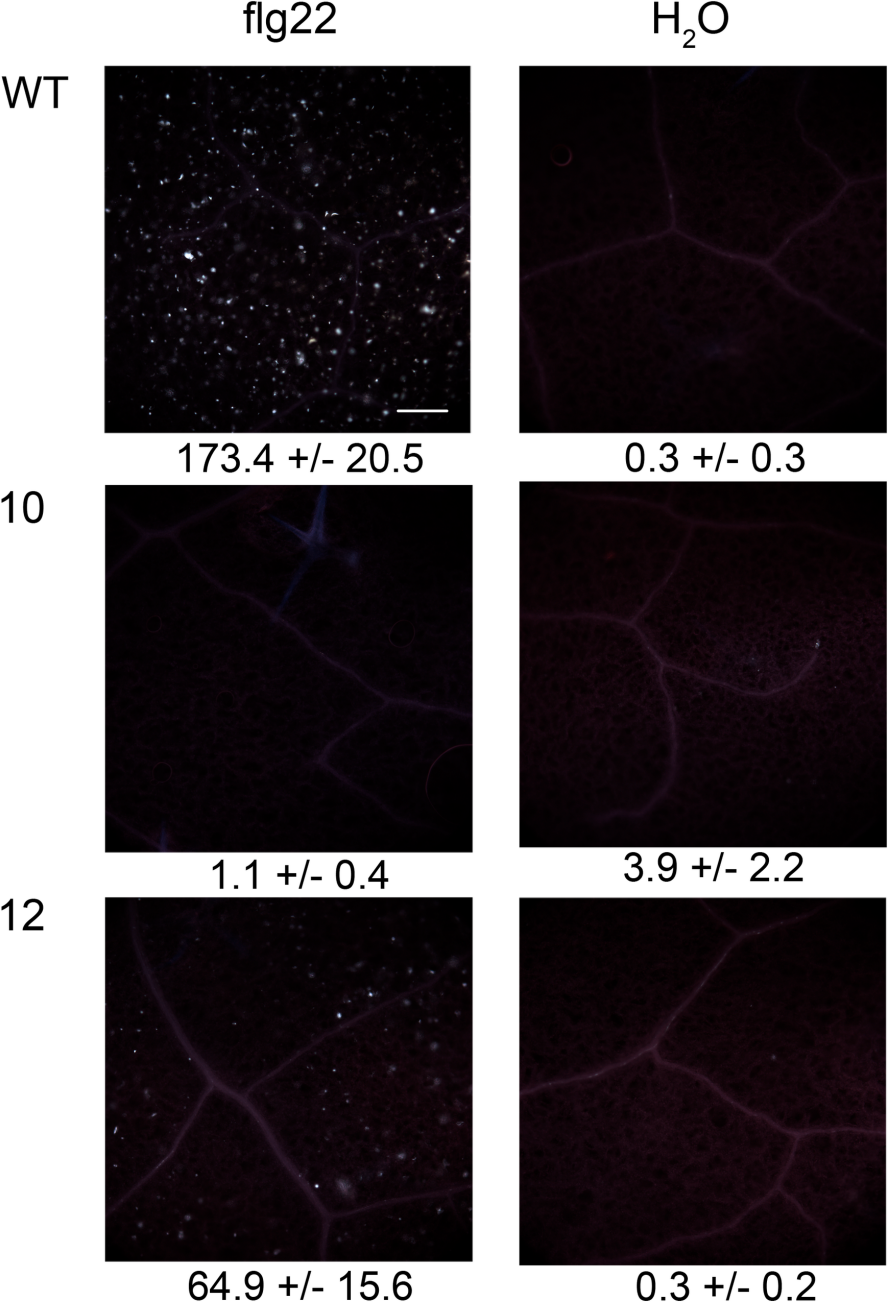
XopB inhibits flg22-triggered callose deposition in transgenic *A*. *thaliana* plants. *A*. *thaliana* wild type and transgenic plants (line 10 and 12) were watered with 10 ml 1% EtOH 18 h before treatment with 1 μM flg22 or deionized water. For each treatment two leaves of three independent plants were infiltrated. After additional 18 h leaves were bleached with ethanol and subsequently stained with aniline blue. Four to six randomly chosen microscopic images per leaf were documented and the number of callose deposits per mm^2^ was counted. Numbers of callose depositions +/- SE are given below the images and are the means of at least 25 values.

A well-known late ROS-dependent response to flg22 treatment is the fortification of cell walls by callose deposition [6,16,21]. A strong accumulation of callose was detected in wild type plants by aniline blue staining 18 h upon flg22 stimulation (Fig 9). In contrast, callose deposition was significantly reduced in the *xopB*-expressing lines 10 and 12 after flg22 treatment (Fig 9).

Together these data suggest that XopB suppresses the basal and PAMP-mediated expression level of peroxidases and *AtRBOHD*. This may lead to a lower ROS production upon PAMP stimulation and disturb downstream signalling pathways thatfor example control callose deposition.

### Deletion of *xopB* in *Xcv* causes higher production of H_2_O_2_ in susceptible pepper plants

**Fig 10:**
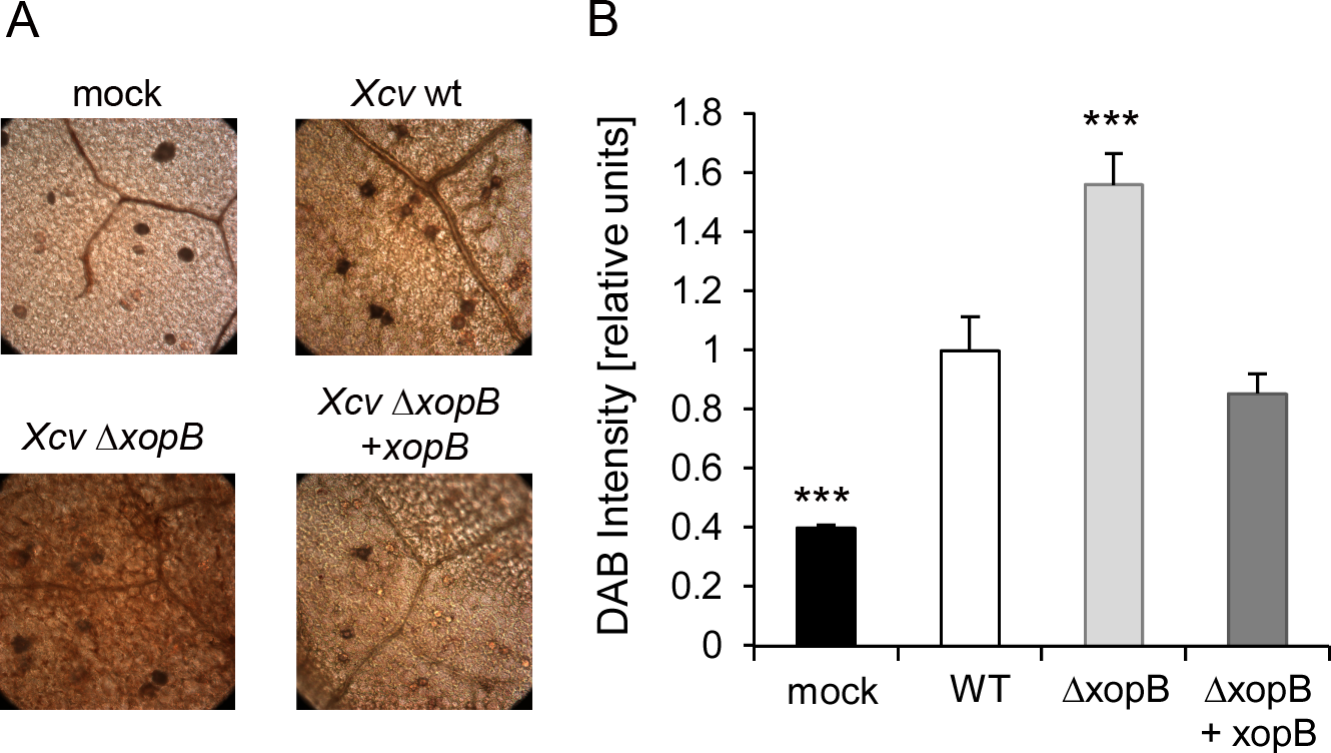
Deletion of *xopB* in *Xcv* leads to higher accumulation of ROS in pepper leaves. Leaves of pepper plants were infiltrated either with *Xcv* wild type (*Xcv* WT), a *xopB* deletion strain (*Xcv* ΔxopB) or a *xopB* deletion strain complemented by *xopB* (*Xcv* Δ*xopB* + *xopB*) at a concentration of 10^9^ cfu ml^-1^. All strains harbour the pBBR1-MCS5 vector. As a control, leaves were infiltrated with 10 mM MgCl_2_. Two leaves of two pepper plants were infiltrated using a needle-less syringe. Three days post infection six 4 cm^2^ leaf discs were taken and stained for ROS using DAB solution. After chlorophyll clearance six randomly chosen microscopic images per leaf disc were documented. **A)** A representative image for each scenario is shown. **B)** Pixel intensities of microscopic images were determined by means of the ImageJ software. Values shown are means +/- SE of 18 images and were presented relative to the mean value of pepper plants infiltrated with *Xcv* wild type strain which was set to one. Statistically significant differences to *Xcv* wild type-infected leaves are indicated by asterisks (***p<0.001).

In order to elucidate whether XopB also inhibits the ROS production during the *Xcv*—pepper interaction, leaves of susceptible pepper plants were infiltrated with *Xcv* wild type, *Xcv* Δ*xopB*, *Xcv* Δ*xopB* + *xopB* and MgCl_2_ (Mock) and stained with 3,3-diaminobenzidine-tetrahydrochloride (DAB) at 3 dpi to monitor H_2_O_2_ accumulation [50]. Compared to mock-inoculated plants, a higher amount of brownish precipitates was visible in pepper leaves after *Xcv* wild type infection indicating an increased pathogen-mediated H_2_O_2_ accumulation (Fig 10A). The accumulation of H_2_O_2_ was even stronger, when plants were infiltrated with the *Xcv* Δ*xopB* strain (Fig 10A). Infection with the complemented strain *Xcv* Δ*xopB* + *xopB* caused a colouration similar to the *Xcv* wild type strain (Fig 10A). Quantification of the images revealed an approximately 50% higher DAB intensity in *Xcv* Δ*xopB-*infiltrated pepper leaves compared to leaves infected with the *Xcv* wild type or the *xopB* complemented strain (Fig 10B). These results corroborate the finding of XopB-mediated inhibition of ROS production.

## Discussion

Transgenic plants have been proven to be a versatile tool to study T3E function and to identify host target processes [51]. For example, expression of the *Pst* DC3000 T3E AvrPto in transgenic *A*. *thaliana* plants provided the first evidence that PTI is suppressed by this effector [52]. Subsequently, numerous further studies contributed to elucidate its molecular mechanisms by using transgenic plants and showed that AvrPto interacts with the kinase domain of different PRRs including FLS2 and thereby inhibits downstream signalling events (e.g. [53,54]).

In order to analyse the role of the *Xcv* effector protein XopB in more detail, we generated transgenic Arabidopsis plants expressing *xopB* in an inducible manner. Expression of *xopB* in Arabidopsis caused severe phenotypic alteration leading eventually to cell death. This is in accordance with earlier observations showing that XopB caused detrimental effects when expressed in plants [37–39]. Although the underlying molecular mechanisms are not clear, these results led us to conclude that this effector modulates cellular processes that finally provoke cell death.

Our recent work suggested that the T3E protein XopB from *Xcv* inhibits the pathogen-stimulated increase of cw-Inv expression and activity in pepper plants [38]. Cw-Inv catalyses the cleavage of the transport sugar sucrose into glucose and fructose and is a key enzyme for supplying sink-organs with carbohydrates [55]. Its expression and activity was shown to be increased in source leaves in response to infection with various pathogens (see [41]). The induction of cw-Inv is thought be crucial to feed an increased metabolic demand of defence responses [56,57]. A fast increase in hexose amounts appeared to be important to mount an effective and successful plant defence. This observation was supported by the finding that heterologous expression of a yeast invertase in either the apoplast or the vacuole of transgenic tobacco plants resulted in high amounts of hexoses and an increased resistance against potato virus Y [56]. Moreover these plants exhibited strongly increased levels of SA and SAG and a higher expression of SA-regulated defence genes [56]. This indicated that an increased invertase activity and the local accumulation of soluble sugars was a prerequisite for the accumulation of SA and an effective defence. Here, we showed that the T3E XopB is involved in modulation of SA/ SAG levels and of SA-dependent genes such as *PR1* and *PR3*. This was seen in the interaction between *A*. *thaliana* and *Pst* as well as between *C*. *annuum* and *Xcv*. Thus, infection of susceptible pepper plants with an *Xcv xopB* deletion strain caused a higher accumulation of SA/SAG and SA-dependent *PR* transcripts compared to infection with an *Xcv* wild type strain. This is in line with the increased cw-Inv expression and activity found after infection of pepper leaves with *Xcv* Δ*xopB* [38]. In *xopB*-overexpressing Arabidopsis plants, however, both the increase in the amount of SA/SAG as well as in *PR* gene expression was clearly lower in response to *Pst* DC3000 infection than in wild type plants indicating that XopB suppresses defence responses during infection with *Pst* DC3000. The latter findings were accompanied by an increased bacterial growth and an accelerated development of disease symptoms suggesting that XopB acts in concert with the T3Es secreted by *Pst* DC3000 to suppress plant defence. *Pst* DC 3000 harbours the T3E HopD1 which is a homolog of XopB. Infection of Arabidopsis plants with an *Pst hopD1* mutant led to a slight, but significant reduction in bacterial growth, but growth of a DC3000 strain was not altered in transgenic plants expressing HopD1 ectopically [58]. However, even though HopD1 and XopB show sequence homology their mode of action might by different since further work by Block et al. [58] revealed that HopD1 reduces ETI responses by targeting the ER-localised transcription factor NTL9, but did not affect PTI, while XopB affects both PTI and ETI [37].

Generally, individual bacterial strains express multiple effectors with apparently distinct and redundant activities which are collectively essential to support their life style, but their effector repertoire shows a high degree of variability [59]. XopB and homologous T3Es from other bacterial strains do not belong to the core group of conserved effector proteins [35], but are rather specific T3Es exploited by some species. Hence, it might conceivable that XopB amend the effector repertoire of *Pst* DC3000 and thereby enhancing its bacterial virulence.

Although the SA-mediated defence responses were activated upon infection of pepper plants with *Xcv* Δ*xopB*, the bacterial growth of this strain was not altered [36,37]. Functional redundancy with other *Xcv* T3E could be the reason why bacterial growth is not significantly affected. Accordingly, Schulze et al. [37] suggested that XopB and XopS fulfil redundant functions based on studies with a double knock out strain. Also a *P*. *syringae pv*. *phaseolicola avrPphD* mutant (AvrPphD is another homolog of XopB) showed no effect on bacterial growth indicating that it is functionally redundant with other T3E [60]. Infection with a single *xopB* deletion *Xcv* strain caused milder disease symptoms ([37]; this study) suggesting that *Xcv* virulence is weakened but obviously not sufficiently enough to affect bacterial growth.

Here, we further analysed how XopB affects PAMP-triggered defence responses. This was stimulated by work of Tsuda et al. [4] who showed that SA levels increase in response to flg22-treatment and that SA is a major component of the PTI signalling cascade. Moreover, Schulze et al. [37] reported that XopB supresses PTI responses like the flg22-mediated activation of the *NHL10* promoter, a well-established marker for PTI-signalling studies [26,28]. Expression of *xopB* also decreased basal activity of *pNHL10* in Arabidopsis protoplasts [37]. Similarly, both basal and flg22-stimulated expression of *NHL10* was reduced in transgenic *A*. *thaliana* plants expressing *xopB*. The flg22-mediated increase in the expression of *AtPHI1* was also abolished in the *xopB*-expressing plants. In contrast, the flg22-stimulated induction of *AtWRKY22* and *AtFRK1* was not significantly altered or even higher in *xopB-*expressing lines as compared to wild type plants. MAPK-dependent as well as CDPK-dependent signalling pathways contribute to the transcriptional re-programming during plant immune responses and both pathways control different groups of target genes [28]. Genes such as *AtFRK1* and *AtWRKY22* are MAPK-specific, while *AtPHI1* is controlled by CDPKs. Other targets like *AtNHL10* are regulated by both CDPK- and MAPK-dependent signalling pathways. The expression pattern of flg22-responsive marker in the *xopB*-expressing Arabidopsis plants genes led us to conclude that XopB alters CDPK- but not MAPK-dependent signalling pathways. This assumption is in accordance with the finding that phosphorylation of MAPKs was not influenced upon flg22 treatment by expression of *xopB* in Arabidopsis protoplasts [37].

Several lines of evidence indicate that PAMP-activation of MAPK may occur independent of the PAMP-triggered ROS production at least during the early stage of PTI (see [61,62]). For instance, the *bik1* mutant is compromised in AtRBOHD-dependent ROS production and callose deposition, but not in flg22-induced MAPK activation [63,64]. The flg22-mediated ROS burst is largely controlled by AtRBOHD [16,17] and was shown to be dependent on the transient influx of calcium ions from the apoplast [7,23]. AtRBOHD activity itself is regulated by calcium binding and by phosphorylation [14,17]. A number of CDPKs have been identified that phosphorylate AtRBOHD [65,66]. Recent work has shown, however, that AtRBOHD becomes phosphorylated by BIK1 upon PAMP stimulation in a calcium-independent manner [12,13]. Although we were not able to directly measure the impact of XopB on Ca^2+^ influx, due to transgene silencing after crossing the *xopB*-lines with the aequorin reporter lines, our results clearly show that expression of XopB strongly suppressed the flg22- stimulated ROS burst and subsequent signalling processes in Arabidopsis. Hence, the transcript abundance of *AtOXI1*, a kinase that is transcriptionally activated by H_2_O_2_ [49] was induced to a lower extent in the *xopB*-expressing Arabidopsis plants than in wild type. Interestingly, *AtRBOHD* transcript abundance increased after flg22-treatment in both wild type and *xopB-*expressing Arabidopsis lines, but the absolute levels were lower in the transgenic plants compared to wild type plants. In addition, the expression of the two apoplastic peroxidases, *AtPRX33* and *AtPRX34*, was down-regulated in *xopB-*expressing control plants compared to wild type and their expression did not increase in response to flg22. Only expression of *PRX33*, but not of *PRX34*, was significantly increased 30 min after flg22 treatment in wild type (Col-0) plants. Results published by Daudi et al. [20] showed an at least 10-fold up-regulation for both genes 2 h after flg22 treatment. Even though these peroxidases were shown to account for about half of the flg22-triggered ROS production [19,20], their expression might be strongly stimulated at later time points after PAMP treatment. Hence, Kadota et al. [15] proposed that the immediate ROS burst after PAMP treatment is entirely dependent on AtRBOHD which then triggers a secondary, late PRX33/34- dependent ROS production.

Transcript accumulation of *AtRBOHD* as well as of *AtPRX33* and *AtPRX34* are positively regulated by H_2_O_2_. Moreover, *AtRBOHD* expression was shown to be dependent on the peroxidase-mediated H_2_O_2_ production [19]. Torres et al. [67] postulated, that NADPH oxidases like AtRBOHD have to be activated by an NADPH oxidase-independent source of ROS, which might be peroxidases. Therefore, it seems conceivable that XopB decreases expression of the apoplastic peroxidases *AtPRX33* and *AtPRX34* which accounts for a reduced basal H_2_O_2_ production and subsequently for a decreased *AtRBOHD* expression. The lower absolute level of *AtRBOHD* may then cause the reduced ROS production upon flg22 stimulation observed in the *xopB*-expressing lines.

The *prx33* and *prx34* as well as the *rbohd* mutant are impaired in PAMP stimulated callose deposition [16,20] supporting the idea that callose deposition is ROS-dependent. Accordingly, a lower callose deposition was observed in the *xopB*-expressing Arabidopsis plants.

Several PAMP-associated genes were found to be down-regulated in un-challenged *prx33* and *prx34* T-DNA insertion lines which led the authors to suggest that a low level of ROS production is required to “preprime” basal defence [20]. Likewise, the observed down-regulation of *AtWRKY22* and *AtNHL10* in un-treated *xopB*-expressing plants might be also related to the low expression of both peroxidases. Moreover, Mammarella et al. [68] reported that transgenic *A*. *thaliana* plants in which expression of *AtPRX33* and *AtPRX34* (and probably other peroxidases) was knocked-down by antisense expression of a cell wall peroxidase cDNA clone from French bean [18] were severely impaired in the induction of SA-responsive genes, such as *AtPR1*. Remarkably, these plants were not more susceptible to infection with a *Pst hrcC*-mutant than wild type plants, although these plants are compromised for a variety of PTI-responses. Similarly, expression of *xopB* caused a down-regulation of the SA-responsive genes *AtPR1* and *AtPR3* and a dampened PTI-response, but did not alter the *in planta* growth of a T3SS-deficient *Pst* ΔTLR1 strain. Since effects caused by XopB expression in *A*. *thaliana* strongly suggest that it supresses PTI responses this finding was unexpected and requires further investigations. Expression of XopB in *A*. *thaliana* inhibited the flg22-induced ROS burst and down-stream signalling events, but the MAPK-dependent signalling was not inhibited or even weakly activated. Together with other, parallel operating PAMP-stimulated signalling pathways this may provide sufficient activation of defence responses to combat propagation of the *Pst* ΔTLR1 strain in *xopB*-expressing lines.

Infection of susceptible pepper plants with a *Xcv xopB*-deletion strain led to a higher ROS accumulation compared to infection with the *Xcv* wild type strain indicating that XopB interferes with ROS accumulation also in the host plants. In pepper plants, CaPO2 was identified as an apoplastic peroxidase pivotal for the oxidative burst [69]. Silencing of *CaPO2* in pepper compromised *PR* gene expression and caused enhanced susceptibility to virulent *Xanthomonas* strains [69]. Moreover, infections of Arabidopsis lines overexpressing *CaPO2* with virulent *Pst* DC3000 resulted in enhanced ROS production [69] confirming the important role of this enzyme. The effect was accompanied by a reduced growth of *Pst* DC3000 and an elevated mRNA level of *PR* genes, including SA-dependent ones [69]. Expression of *xopB* in *A*. *thaliana* plants caused similar molecular and biochemical changes as observed in *prx33/34* mutants, while infection of pepper plants with an *Xcv xopB* deletion strain led to phenotypes comparable to plants expressing *CaPO2* suggesting that XopB may interfere with expression and/ or activity of peroxidases.

How can the observed effects by XopB be linked to its localisation in vesicle-like structures described by Schulze et al. [37]? Vesicle trafficking emerged as an important means of plant defence contributing to the correct localisation of PAMP receptors [70]. In more detail, endocytosis was recently shown to be involved in PAMP-stimulated ROS production [70,71] and regulation of AtRBOHD activity [72]. XopB altered the ROS production during PAMP-treatment and *Xcv* infection, but appeared not to affect MAPK signalling. Other T3E proteins have been described which also impair the ROS burst, but show normal MAPK signalling such as AvrAC and AvrPphB [63,64]. AvrAC is an uridyl transfrase that reduces BIK activity, while AvrPphB is a cysteine protease cleaving BIK1 and other PBS1-like kinases. Host proteins targeted by XopB are so far not known, but need to be identified in future work to unravel the molecular and cellular control mechanisms exerted by XopB. Our current data suggest that XopB inhibits peroxidase mediated ROS-production. How this is related to its proposed inhibitory effect on vesicle transport (or endocytotic processes) needs to be learned from further work.

## Material and Methods

### Generation of transgenic Arabidopsis plants

Stable transformation of *Arabidopsis thaliana* Col-0 plants of the EtOH::*xopB* construct [38] was performed by *Agrobacterium-*mediated gene transfer as described previously [73].

For selection of transgenic lines, T1 seeds were surface-sterilized and sown onto Murashige-Skoog medium supplemented with 50 μg ml^-1^ kanamycin. Expression of the transgene was confirmed by northern blotting. Numbers of T-DNA insertions were determined by southern blotting.

### Plant material and growth conditions

*A*. *thaliana* plants (T3 or T4 generation of transgenic lines) were sown on soil and kept for 2–3 days at 4°C in darkness to synchronize germination. Subsequently, seedlings were grown for two weeks under short day conditions (8 h of light, 22°C; 16 h darkness, 20°C) in phytotrons (CLF, Wertingen, Germany) before transfer into individual pots. Further cultivation was done in a plant climate chamber (Plant Master PGR 3045, CLF Wertingen, Germany) under short day conditions with approximately 100 μmol quanta m^-2^ s^-1^ light and 60% relative humidity. Expression of *xopB* was induced by watering individual pots with 10 ml 1% (v/v) ethanol and was confirmed by western blotting using a XopB-specific antibody [38] in every experiment.

Pepper plants (*Capsicum annuum* cv. Early Cal Wonder (ECW)) and *Nicotiana benthamiana* plants were cultivated in a greenhouse at 26°C or 25°C, respectively, with 16 h supplemental light (150–200 μmol quanta m^-2^ sec^–1^) and 60% relative humidity and 22°C during 8 h of darkness.

### Bacterial strains and infection experiments

Bacterial strains used in this study are described in S2 Table. *Pseudomonas syringae* pv. *tomato* (*Pst*) strain DC3000 *Pst* and *Xanthomonas campestris* pv. *vesicatoria* (*Xcv*) 85–10 strains were cultivated at 28°C on nutrient-yeast-glycerol (NYG) medium supplemented with appropriate antibiotics. Antibiotics were added to the media at following final concentrations: kanamycin, 50 μg ml^-1^; rifampicin, 100 μg ml^-1^ and gentamycin 15 μg ml^-1^. Infection of five week old *A*. *thaliana* leaves with *Pst* DC3000 was carried out as follows: *Pst* strains were plated from DMSO cryo-stocks on NYG plates supplemented with appropriate antibiotics and incubated at 28°C for two to three days. Bacterial cells were further cultivated in liquid media, washed and adjusted with sterile 10 mM MgCl_2_ to desired cell densities. For *in planta* growth studies, virulent and TTSS-deficient *Pst* strains were syringe-infiltrated with a density of 5*10^5^ cfu ml^-1^or 10^6^ cfu ml^-1^, respectively. Bacterial titres of *Pst in planta* were determined as described in Sonnewald et al. [38]. For SA quantifications and qPCR studies, *Pst* DC3000 was syringe-infiltrated with a bacterial density of 2.5*10^6^ cfu ml^-1^. *Xcv* infection of pepper leaves was performed as described previously [38].

### Flg22-treatment

Five week old *A*. *thaliana* wild type or transgenic plants were infiltrated with 1 μM flg22 peptide or with deionized water. For analysis of gene expression leaf material was harvested 30 or 60 min after infiltration, frozen in liquid nitrogen and stored at -80°C until RNA extraction.

### Isolation of genomic DNA and Southern blotting

Genomic DNA was isolated from 5 g leaf material of 5 week old Arabidopsis plants. Restriction enzyme digestion with *EcoR*I and southern blot analysis was performed as described previously [74].

### RNA isolation and quantitative RT-PCR

Isolation of total RNA was performed as described in [75]. Copy DNA was usually synthesized from 2 μg total DNaseI-treated RNA using Oligo dT30 primers. Quantitative real time PCR (qPCR) was essentially performed as described by [76]. For each primer pair the efficiency was determined which was between 96% -113%. A melting analysis was performed at the end of each run to ensure that unique products were formed. Relative expression values of target genes were calculated using the Pfaffl method [77]. Fold changes in gene expression were calculated according to Livak and Schmittgen [78]. The values were normalized to elongation factor 1 alpha (*EF1 alpha*) from pepper and tubulin 4 (*TUB4*) from Arabidopsis, respectively. For semi-quantitative RT-PCR, cDNA was diluted 1:10 and subjected to standard PCR protocol. Primers used for qPCR and semi-quantitative PCR analyses are listed in S3 Table.

### Western blotting

Leaf discs (0.5 cm^2^) were homogenized and processed as described previously [38]. Proteins were detected by using an anti-XopB antibody by means of chemiluminescence.

### Determination of SA and SA glucosides

Extraction of free SA and SA glucosides was performed as described previously [79]. HPLC-based separation of SA and SA glucosides was performed on a Dionex Summit system (P680, ASI-100, TCC-100, RF-2000) equipped with a Phenomenex Luna Security Guard C18 column (4.0 * 3.0 mm) followed by a 5-mm Luna C18(2) reverse-phase column (250 * 4.6 mm) as described by Voll et al. [80].

### Luminol-based detection of ROS

Leaf discs (0.25 cm^2^) were incubated overnight at 22°C in the dark in a 96-well micro titer plate filled with 0.2% EtOH (to induce expression in transgenic *A*. *thaliana* plants) or deionized water (for *N*. *benthamiana*). The solution was then substituted by 200 mM sodium phosphate buffer, pH 8.0 and a luminol assay solution consisting of 0.2 mM luminol (Sigma, A8511) and 0.01 mg ml^-1^ type VI horseradish peroxidase (Sigma, P8375). The micro titre plates were placed in the plate reader (Mithras LB 940, Berthold, Bad Wildbad, Germany) and background signals were detected with 1 sec integration time per well for 5 min. After manual addition of 1 μM flg22 the luminescence was recorded with 1 sec integration time for 45 min. For each genotype the mean background value was calculated and subtracted from flg22-triggered luminescence values.

### Detection of callose deposition

*A*. *thaliana* Col-0 wild type and transgenic plants were watered with 10 ml 1% EtOH 18 h before treatment with 1 μM flg22 or deionized water. Two leaves of three independent plants were infiltrated for each treatment. After 18 h leaves were de-stained in 96% EtOH and further processed as described by [81]. Epifluorescence microscopy was conducted with a Leica DMR microscope using UV excitation and a DAPI emission filter. Six randomly chosen areas per leaf were documented and callose depositions per mm^2^ were counted.

### Transient expression of *xopB* in *N*. *benthamiana*

For transient expression of *xopB*, the ORF was amplified with the primers 5’-xopB (5’-aggatcctctagaatgaaggcagagctcacacgatccca-3’) and 3’-xopB (5’-gtcgaccggctcaggcgcgggttggtg-3’) and inserted into expression vector pBinAR under the control of the 35S promoter [82] via BamHI and SalI restriction sites (restrictions sites in primer sequences are underlined). For infiltration of *N*. *benthaniama* leaves, *Agrobacterium tumefaciens* C58C1 strains were infiltrated into the abaxial leaf site of 4- to 6-week-old plants. *A*. *tumefaciens* strains harbouring *xopB*, the silencing suppressor p19 [83] or the empty vector were cultivated overnight at 28°C in the presence of appropriate antibiotics. The cells were harvested by centrifugation, re-suspended in infiltration buffer (10 mM MgCl_2_, 10 mM MES pH 5.6 and 100 μM acetosyringone), adjusted to oD_600_ of 0.4. Cell suspensions containing either the *xopB* expression construct or the empty vector were mixed with p19 (1:1 ratio). Samples for assaying ROS production and protein detection were taken 48h after *Agro*-infiltration.

### Histochemical detection of ROS

Leaf discs (4 cm^2^) of infected pepper plants were placed into a solution containing 1 mg ml^-1^ 3.3´-diaminobenzidine-tetrahydrochloride, pH 3.8 (DAB, Applichem, A0596). After incubation in the dark for 18 h the DAB solution was replaced by 96% EtOH and boiled at 80°C until the chlorophyll was completely removed. Leaf discs were mounted in 40% glycerol and observed under a Leica DMR microscope using bright field. For quantification of the pixel intensities, 6 randomly chosen areas per leaf discs were recorded resulting in 18 images per condition. Quantification with ImageJ was essentially conducted as described by Rodríguez-Herva et al. [84]. Images with a 200-fold magnification were used. Pixel intensities of samples taken *Xcv* wildtype infected pepper leaves served as a calibrator and were arbitrary set to one.

## Supporting Information

S1 Fig Characterisation of transgenic *xopB*-expressing *Arabidopsis thaliana* lines. **A)** Schematic representation of the T-DNA harbouring the EtOH::*xopB* expression cassette. The transcriptional regulator AlcR from *Aspergillus nidulans* is expressed under control of the cauliflower mosaic virus 35S promoter (35S) and the nopaline synthase terminator (NOS) from *Agrobacterium tumefaciens*, while expression of the type III effector *xopB* from *Xanthomonas campestris* pv. *vesicatoria* (cloned in 5´-3´ orientation) is driven by a modified promoter of the alcohol dehydrogenase AlcA from *Aspergillus nidulans* and 35S terminator (35S T). Numbers indicate base pairs number of the corresponding elements. The right border and the kanamycin resistance cassette of the T-DNA are not shown. The depicted T-DNA construct has three *EcoR*I sites within the T-DNA sequence and one additional *EcoR*I site at base pair position 463 of *xopB*. **B)** Selection of transgenic lines by northern blotting. Total RNA was extracted from different transgenic lines upon floating of detached leaves with 0.2% EtOH in the dark overnight. Twenty μg of total RNA was separated in a formaldehyde-containing agarose gel and blotted onto nitrocellulose membrane. Nine *xopB* expressing transgenic T1 lines from *A*. *thaliana* could be identified using a *xopB* specific ^[^^32^^]^P-labelled probe. After stripping the same membrane was incubated with a probe specific for the small subunit of *RubisCO* (*RbcS*). **C)** Southern blot analysis of lines 10, 12, 27 and 16. Ten μg genomic DNA of T3 or T4 plants were digested with *EcoR*I and analysed by Southern blotting. The first 463 base pairs of *xopB* were used as probe for the hybridisation. EtOH::*xopB* lines 10 and 12 harbour one T-DNA insertion, whereas lines 27, 16–6 and 16–8 have multiple insertions.
(TIF)

S2 Fig XopB inhibits flg22-triggered ROS production in *N*. *benthamiana*. *Agrobacterium tumefaciens* strains harbouring either *xopB* (in pBinAR) or the empty vector were infiltrated together with the silencing suppressor p19 into *N*. *benthamiana* plants. Two days post infiltration leaf discs were sampled to analyse **A)** flg22-stimulated ROS production and **B)** XopB protein accumulation by western blotting. Experimental details are described in “Material and Methods”. Similar results were obtained in three independent experiments.
(TIF)

S1 Table Fold induction (flg22- vs. H_2_O-treatment) of target gene expression in wild type, *xopB*- expressing *Arabidopsis* lines (10, 12) and *fls2* mutant. Values were calculated according to [78] and are the mean ± SD of three independent replicates. Significant differences (p-value ≤ 0.05) compared to wild type are indicated by * and corresponding values are highlighted in bold.
(DOCX)

S2 Table Bacterial strains used in this study. (XLSX)

S3 Table List of primer sequences used for PCR. (XLSX)
