# Supplementary figures and images for: The Xanthomonas campestris pv. vesicatoria Type-3 Effector XopB Inhibits Plant Defence Responses by Interfering with ROS Production
Source: PLoS One. 2016 Jul 11;11(7):e0159107. doi: 10.1371/journal.pone.0159107 (PMC4939948; doi:10.1371/journal.pone.0159107)

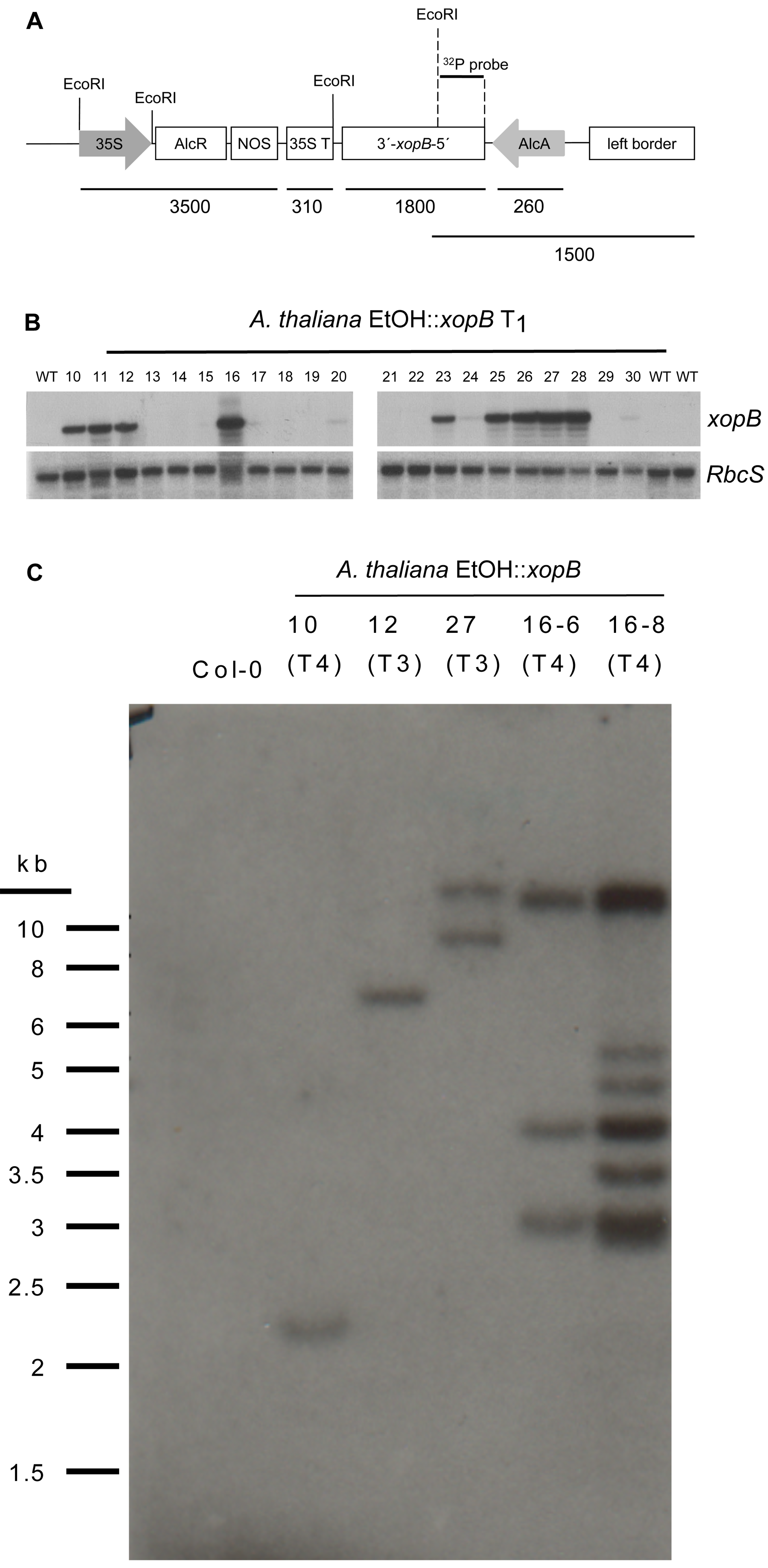

Supplement: S1 Fig — A) Schematic representation of the T-DNA harbouring the EtOH::xopB expression cassette. The transcriptional regulator AlcR from Aspergillus nidulans is expressed under control of the cauliflower mosaic virus 35S promoter (35S) and the nopaline synthase terminator (NOS) from Agrobacterium tumefaciens, while expression of the type III effector xopB from Xanthomonas campestris pv. vesicatoria (cloned in 5´-3´ orientation) is driven by a modified promoter of the alcohol dehydrogenase AlcA from Aspergillus nidulans and 35S terminator (35S T). Numbers indicate base pairs number of the corresponding elements. The right border and the kanamycin resistance cassette of the T-DNA are not shown. The depicted T-DNA construct has three EcoRI sites within the T-DNA sequence and one additional EcoRI site at base pair position 463 of xopB. B) Selection of transgenic lines by northern blotting. Total RNA was extracted from different transgenic lines upon floating of detached leaves with 0.2% EtOH in the dark overnight. Twenty μg of total RNA was separated in a formaldehyde-containing agarose gel and blotted onto nitrocellulose membrane. Nine xopB expressing transgenic T1 lines from A. thaliana could be identified using a xopB specific [32]P-labelled probe. After stripping the same membrane was incubated with a probe specific for the small subunit of RubisCO (RbcS). C) Southern blot analysis of lines 10, 12, 27 and 16. Ten μg genomic DNA of T3 or T4 plants were digested with EcoRI and analysed by Southern blotting. The first 463 base pairs of xopB were used as probe for the hybridisation. EtOH::xopB lines 10 and 12 harbour one T-DNA insertion, whereas lines 27, 16–6 and 16–8 have multiple insertions. (TIF) [file pone.0159107.s001.tif]

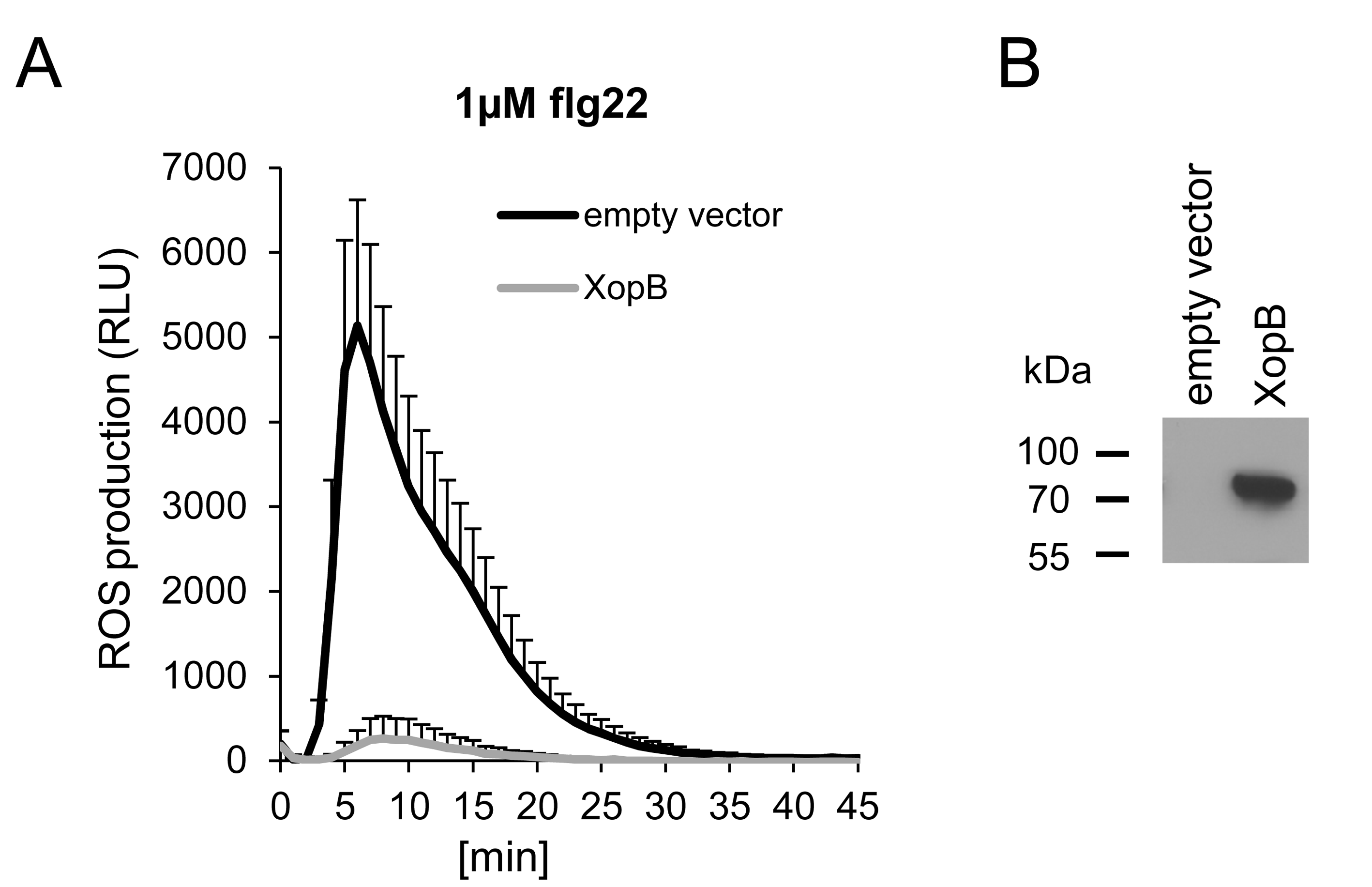

Supplement: S2 Fig — Agrobacterium tumefaciens strains harbouring either xopB (in pBinAR) or the empty vector were infiltrated together with the silencing suppressor p19 into N. benthamiana plants. Two days post infiltration leaf discs were sampled to analyse A) flg22-stimulated ROS production and B) XopB protein accumulation by western blotting. Experimental details are described in “Material and Methods”. Similar results were obtained in three independent experiments. (TIF) [file pone.0159107.s002.tif]
