# Supplementary material for: The Xanthomonas campestris pv. vesicatoria Type-3 Effector XopB Inhibits Plant Defence Responses by Interfering with ROS Production
Source: PLoS One. 2016 Jul 11;11(7):e0159107. doi: 10.1371/journal.pone.0159107 (PMC4939948; doi:10.1371/journal.pone.0159107)
Supplement: S1 Table — Values were calculated according to [78] and are the mean ± SD of three independent replicates. Significant differences (p-value ≤ 0.05) compared to wild type are indicated by * and corresponding values are highlighted in bold. (DOCX) [file pone.0159107.s003.docx]

Supplemental Table 1: Fold induction (flg22- vs. H_2_O-treatment) of target gene expression in wild type, *xopB*- expressing *Arabidopsis* lines (10, 12) and *fls2* mutant. Values were calculated according to [78] and are the mean ± SD of three independent replicates. Significant differences (p-value ≤ 0.05) compared to wild type are indicated by * and corresponding values are highlighted in bold.

| Target gene/ genotype | **WT** | **10** | **12** | ***fls2*** |
| --- | --- | --- | --- | --- |
|  | mean ± SD | mean ± SD | mean ± SD | mean ± SD |
| *NHL10* | 3.8 ± 0.47 | **0.83** ± 0.31* | **1.7** ± 0.35* | **0.99** ± 0.37* |
| *WRKY22* | 14.6 ± 1.7 | 13.8 ± 4.9 | **25.8** ± 6.5* | **1.3** ± 0.54* |
| *FRK1* | 15.4 ± 5.9 | 19.6 ± 6.6 | **45.2** ± 11.6* | **0.75** ± 0.06* |
| *PhI1* | 2.6 ± 0.37 | 0.88 ± 0.31* | 0.97 ± 0.25* | **0.25** ± 0.06* |
| *Oxi1* | 9.4 ± 2.6 | **3.2** ± 0.10* | **2.7** ± 1.6* | **1.0** ± 0.66* |
| *RbohD* | 2.7 ± 0.70 | 2.6 ± 0.48 | 4.0 ± 1.3 | **1.5** ± 0.54* |
| *PRX33* | 4.6 ± 0.2 | **1.3** ± 0.3* | **1.6** ± 0.51* | **1.2** ± 0.26* |
| *PRX34* | 1.6 ± 0.67 | 1.3 ± 0.28 | 1.4 ± 0.23 | 1.3 ± 0.35 |
